# Transition Metal High‐Entropy Nanozyme: Multi‐Site Orbital Coupling Modulated High‐Efficiency Peroxidase Mimics

**DOI:** 10.1002/advs.202303078

**Published:** 2023-10-23

**Authors:** Jianxing Feng, Xuewei Yang, Ting Du, Liang Zhang, Pengfei Zhang, Junchen Zhuo, Linpin Luo, Hao Sun, Yaru Han, Lizhi Liu, Yizhong Shen, Jianlong Wang, Wentao Zhang

**Affiliations:** ^1^ College of Food Science and Engineering Northwest A&F University 22 Xinong Road Yangling Shaanxi 712100 China; ^2^ Department of Chemical Engineering Columbia University New York NY 10027 USA; ^3^ Department of Anesthesiology Division of Critical Care Medicine Boston Children's Hospital Harvard Medical School Boston MA 02115 USA; ^4^ School of Food & Biological Engineering Key Laboratory for Agricultural Products Processing of Anhui Province Hefei University of Technology Hefei 230009 China

**Keywords:** density functional theory calculations, high‐entropy alloy, multifunctional applications, nanozyme, peroxidase‐like

## Abstract

Strong substrate affinity and high catalytic efficiency are persistently pursued to generate high‐performance nanozymes. Herein, with unique surface atomic configurations and distinct d‐orbital coupling features of different metal components, a class of highly efficient MnFeCoNiCu transition metal high‐entropy nanozymes (HEzymes) is prepared for the first time. Density functional theory calculations demonstrate that improved d‐orbital coupling between different metals increases the electron density near the Fermi energy level (*E*_F_) and shifts the position of the overall d‐band center with respect to *E*_F_, thereby boosting the efficiency of site‐to‐site electron transfer while also enhancing the adsorption of oxygen intermediates during catalysis. As such, the proposed HEzymes exhibit superior substrate affinities and catalytic efficiencies comparable to that of natural horseradish peroxidase (HRP). Finally, HEzymes with superb peroxidase (POD)‐like activity are used in biosensing and antibacterial applications. These results suggest that HEzymes have great potential as new‐generation nanozymes.

## Graphical abstract

Limited catalytic efficiency and poor selectivity are bottlenecks in the development of nanozymes. This work integrates state‐of‐the‐art high‐entropy alloys with currently reported intrinsic enzyme activity sites to design a class of high‐efficiency transition metal nanozymes. This unique design strategy advances the systematic understanding of the structure‐performance relationship between the electronic structure and catalytic activity of nanozymes.

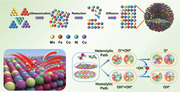

## 1 Introduction

Nanozymes are explicitly defined as a collection of nanomaterial‐based artificial enzyme mimics, and they have gradually evolved as alternatives to natural enzymes due to their outstanding environmental tolerance, recyclability, and long‐term stability.^[^ ^1^ ^]^ Since the first accidental discovery of the inherent POD‐like activity of magnetic Fe_3_O_4_ nanoparticles by Yan and coworkers in 2007, single‐ or multicomponent nanoscale noble metals, metal oxides, metal sulfides, alloys, carbon materials, and metal‐organic framework catalysts have emerged and been widely applied in therapeutics, tissue engineering, and biosensing.^[^ ^2^, ^3^, ^4^, ^5^ ^]^ On the microstructural side, the flexibility in active site element selection and the diversity of binding types and modes of nanozymes also ensure their remarkable modifiability and infinite potential in multiple domains.^[^ ^6^, ^7^, ^8^, ^9^ ^]^ However, despite the tremendous progress that has been achieved thus far, limited catalytic efficiencies and poor selectivities, which mainly emanate from the low intrinsic activities and the ambiguous catalytic mechanisms, have created bottlenecks for the development of nanozymes.^[^ ^7^, ^10^, ^11^, ^12^ ^]^ Of note, multimetallic nanozymes have recently attracted considerable attention due to their unique cocktail effects. The electronic interactions of the metal components may also lead to unexpected properties while achieving nonlinear enhancement of the intrinsic performance.^[^ ^11^, ^13^, ^14^, ^15^ ^]^ However, most conventional design concepts involving doping, heterojunctions, or a combination of both are trial‐and‐error strategies, and the limited component space for these empirical approaches together with the lack of theoretical guidance have hindered diversification and catalytic performance improvements of the nanozymes.^[^ ^8^ ^]^ Therefore, guided by predictive models of catalytic activity, such as the d‐band model and *e*_g_‐occupancy, the development of multimetallic nanocatalytic platforms with a wide range of elements would create a new revolution in nanozymes.^[^ ^7^, ^16^, ^17^ ^]^

To resolve these issues, we introduced the concept of high entropy into the development of high‐performance nanozymes, which integrate state‐of‐the‐art high‐entropy alloys with intrinsic enzyme‐like active sites. High‐entropy alloys (HEAs) provide effective countermeasures to critical catalyst challenges due to their high tunability and flexibilities derived from the multidimensional constituent space.^[^ ^18^, ^19^, ^20^ ^]^ Recently, considerable effort has been devoted to highlighting the superior electrocatalytic performance and stabilities of HEAs in redox reactions.^[^ ^21^, ^22^, ^23^, ^24^ ^]^ Unlike conventional alloys, HEAs have defined content boundaries (5–35%) for the principal elements (≥ 5 species), but no particular element dominates.^[^ ^25^, ^26^ ^]^ In an ideal HEA, the formation enthalpy of the compound is overcome by the dramatic increase in configurational entropy induced by the mixing of the multiple components, facilitating the formation of stable single‐phase solid solutions rather than intermetallic compounds.^[^ ^25^, ^27^, ^28^ ^]^ Accordingly, they tend to form multicomponent single phases with face‐centered cubic (FCC), body‐centered cubic, and hexagonal‐close‐packed structures, in which the internal atoms are randomly distributed.^[^ ^18^, ^29^ ^]^ Therefore, HEAs are also referred to as complex solid solutions.^[^ ^30^ ^]^ In theory, confinement of the atoms with disparate properties and atomic sizes in the same lattice would lead to noticeable lattice distortions and synergistic effects, which normally generate a definite structure‐property relationship for the catalyst.^[^ ^19^ ^]^ More specifically, the abundant and varied atomic sites in HEAs optimize the geometric and electronic configurations of the reactive surface, which in turn affects the adsorption energies of substrates or intermediates and ultimately the catalytic activity. In addition, the strategy of tuning HEAs to nanostructures enables amplification of the size effect and consequently improvement of their catalytic performance.^[^ ^20^, ^31^, ^32^ ^]^ As such, mainstream nano‐HEAs with tailored components and structures are of interest in energy‐related fields, especially in electrocatalysis, due to their superior catalytic performance and stability.^[^ ^23^, ^24^, ^33^ ^]^ Recent studies have highlighted the emergence of high‐entropy materials with POD‐like or oxidase (OXD)‐like activity, which can be exploited for efficient nanozyme‐based antitumor and antibacterial therapies.^[^ ^34^, ^35^ ^]^ Additionally, nanotransition metals are important multimetallic nanozymes due to their easy accessibility, low costs, and excellent catalytic performance. Theoretically, the relatively explicit and easy‐to‐tune d‐band structures of transition metals are potentially advantageous for the design and structure‐property relationship studies of HEzymes, which suggests that effort is still needed to fill this research gap.^[^ ^11^, ^14^, ^15^ ^]^

In this work, we report the first transition metal high‐entropy POD mimic with strong substrate affinity and high catalytic efficiency. The proposed HEzymes contain five transition metals with similar atomic radii, which frequently serve as the catalytic active sites of redox nanozymes, including manganese, iron, cobalt, nickel, and copper.^[^ ^1^, ^3^ ^]^ DFT calculations offer insight into the extraordinary POD‐like activity of the HEzymes. The strong interactions among the *d* electrons of the elements in the HEA NPs optimize the electronic distribution around the E_F_of the bulk material, which enhances the adsorption of intermediates at the surface‐active sites while increasing the efficiency of electron transfer during the catalytic process, thereby enhancing the performance of the nanocatalyst. As a proof of concept, the HEsymes were used as biosensors and in biomedicine. In addition to guaranteeing both selectivity and accuracy of the colorimetric platform for the target, the HEzymes also achieved efficient elimination of multidrug‐resistant bacteria and biofilm eradication by triggering ROS outbreaks. Our findings advance the systematic understanding of the structure‐property relationships between the electronic structure and catalytic performance of nanozymes and provide an original paradigm for engineering high‐performance HEzymes.

## 2 Result

### 2.1 Syntheses and Characterizations of MnFeCoNiCu HEzymes

**Figure 1:**
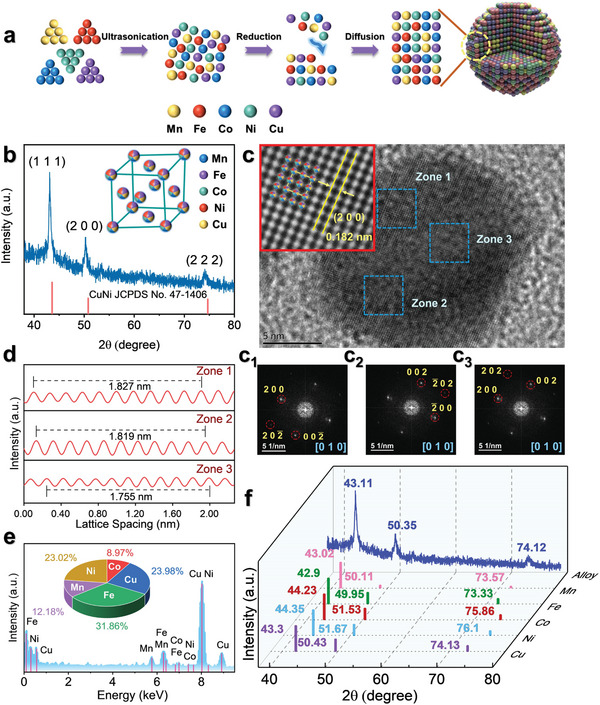
Synthesis and characterizations of the MnFeCoNiCu HEA NPs. a) Schematic illustration showing the formation process of the HEA NPs. b) XRD pattern and crystal structure (inset). c) HRTEM image. The inset diagram shows the atomic arrangement. c_1‐_‐c_3_ are the corresponding FFT patterns along the [010] zone axis for Zone 1‐Zone 3. d) Integrated intensity profiles for pixels in Zone 1–3 of the HRTEM image of the HEA NPs. e) HAADF‐STEM‐EDS spectrum and metallic element contents obtained by ICP‒OES. **f** HEA NPs versus pure metals in the XRD patterns.

MnFeCoNiCu HEA NPs were fabricated via a low‐temperature oil phase synthetic strategy.^[^ ^23^, ^33^ ^]^ Manganese(II) acetylacetonate (Mn(acac)_2_), iron(III) acetylacetonate (Fe(acac)_3_), cobalt(III) acetylacetonate (Co(acac)_3_), nickel(II) acetylacetonate (Ni(acac)_2_) and copper acetylacetonate (Cu(acac)_2_) were used as the metal precursors, glucose as the reductant, and cetyltrimethylammonium bromide (CTAB) and oleylamine (OAm) as solvents and structure inducers. A brown, finely dispersed mixture of the raw materials was formed through ultrasonication. As the temperature of the oil bath increased, the mixture turned black, possibly due to the coordination and coreduction of the five metals. Subsequently, the single‐phase HEA was formed after diffusion and rearrangement of the atoms (**Figure** **1**a).^[^ ^33^ ^]^ To reveal the crystal lattice parameters and morphology of HEA NPs, X‐ray diffraction (XRD), transmission electron microscope (TEM), high‐resolution TEM (HRTEM), and scanning electron microscopy (SEM) were used to characterize the prepared HEA NPs. As shown in Figures S1 and S2 (Supporting Information), the synthesized HEA were nanoparticles with an average diameter of 20.55 nm. The XRD pattern for the HEA NPs indicated a FCCstructure, and the peaks at 44.10° and 50.33° were attributed to the (111) and (200) facets of CuNi (JCPDS No. 47–1406), respectively (Figure 1b). A representative HRTEM image of the HEA NPs exhibited an average lattice spacing of 0.182 nm, which corresponded to the (200) facet of CuNi, illustrating a single FCC phase in agreement with the XRD result (Figure 1c). Of note, the fast Fourier transform (FFT) patterns of selected regions are displayed in Figure c_1_‐c_3_ and suggest that the alloy had a (010)‐oriented FCC structure. The average lattice spacings determined from the FFT patterns of selected regions varied from 1.755 to 1.827 Å, which implied lattice distortions in the alloy phase (Figure 1d; Figure S3, Supporting Information).^[^ ^36^ ^]^ To investigate the effects of the reagents on the morphology of the alloy, TEM images of different products prepared under various reagent combinations are presented in Figures S4–S7 (Supporting Information). Clearly, CTAB or STAB serves to direct the alloy to form uniformly dispersed nanoparticles, while glucose functions as the reductant to control the size and morphology of the alloy.^[^ ^33^, ^37^ ^]^ Furthermore, HEA NPs fabricated with CTAB display a more uniform distribution of size dimensions compared to those prepared with STAB, resulting in a more consistent shape and morphology distribution.

The energy dispersive spectroscopy (EDS) maps and line scans of the HEA NPs showed that the five metallic elements were homogeneously distributed in the nanoparticles, which provides evidence for the successful synthesis of the MnFeCoNiCu quinary HEA NPs (Figure 1e; Figures S8 and S9a, Supporting Information). Likewise, the HEA NPs had 2θ values for the major peaks in the XRD patterns that were consistent with those for the pure constituent metals forming FCC phases (Figure 1f). Obviously, the diffraction peaks for the HEA NPs were broadened, and their positions were slightly shifted compared with those of the pure metals, confirming the formation of the nanometer‐scale alloy.^[^ ^24^ ^]^ According to the quantitative results of the inductively coupled plasma optical emission spectroscopy (ICP‒OES) and X‐ray photoelectron spectra (XPS) (Figure 1e; Figure S9b, Supporting Information), the atomic ratio for Mn, Fe, Co, Ni and Cu was 12: 32: 9: 23: 24. The configurational entropy of MnFeCoNiCu nanoparticles was calculated as 1.52 R, surpassing the boundary of 1.5 R separating high‐entropy and medium‐entropy alloys. Therefore, MnFeCoNiCu nanoparticles are classified as members of high‐entropy materials. Then, XPS was employed to analyze the surface states of the HEA NPs. Figure S10 (Supporting Information) displays the 2p XPS spectra obtained for the 2p edges of Mn, Fe, Co, Ni, and Cu. Of note, the five metals in the HEA NPs all showed mixed valence states, including metallic and oxidized states.^[^ ^23^, ^37^ ^]^ The Fourier transform‐infrared (FT‐IR) spectrum of the HEA NPs showed peaks at 1485.19 and 3016.66 cm^−1^, which arose from CTAB, and these disappeared or were significantly attenuated, indicating that the residual surfactant on the surfaces of the particles had been removed (Figure S11, Supporting Information). In addition, the test photographs illustrated that the prepared HEA NPs were dispersed and remained stable during storage in aqueous solutions (Figure S12, Supporting Information).

### 2.2 Nanozyme Catalytic Performance of the HEzymes

**Figure 2:**
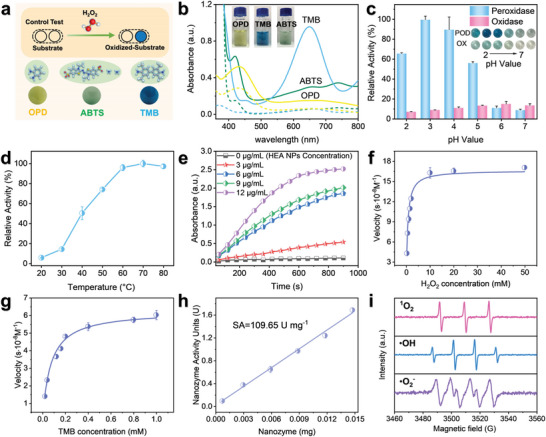
Peroxidase‐like activity and mechanism of the HEA NPs. a) Schematic diagram of the POD‐like activity evaluations of the HEA NPs with OPD, ABTS, and TMB. b) UV‒vis absorption spectra and visual colors of different chromogenic reactions (OPD: yellow line, ABTS: green line, and TMB: blue line). c) OXD‐like and POD‐like activities of the HEA NPs at different pH values. d) POD‐like activity of the HEA NPs at different temperatures. Data are presented as the mean ± SD (standard deviation) (*n =* 3 independent samples). e) Time‐dependent absorbance spectra with different concentrations of HEA NPs. f–g) Michaelis‒Menten curves of the HEA NPs with different concentrations of H_2_O_2_ and TMB, respectively. The data are presented as the mean ± SD (*n =* 3 independent samples). h) Specific activity of the HEA NPs. i) ESR spectra showing ^1^O_2_, ^•^OH and ^•^O_2_^−^ formed from H_2_O_2_. The data are presented as the mean ± SD (*n =* 3 independent samples).

Inspired by the structure and catalytic pathway of natural HRP, the development of POD‐like enzymes involving transition metal elements as protagonists has proven to be efficient and economical.^[^ ^38^ ^]^ Herein, the enzyme‐like activity of HEA NPs was systematically investigated by using 3,3′,5,5′‐tetramethylbenzidine (TMB), TMB as the chromogenic substrate. As shown in **Figure** **2**a, transition metal‐based POD mimics catalyzed the decomposition of H_2_O_2_, resulting in the color rendering of the chromogenic substrates. Specifically, oxidation reaction of 2,2′‐Azino‐bis (3‐ethylbenzothiazoline‐6‐sulfonic acid) diammonium salt (ABTS), o‐phenylenediamine (OPD) and TMB were catalyzed by the HEA NPs in the absence of H_2_O_2_, and the typical peaks (ABTS: 415 nm, OPD: 431 nm, TMB: 652 nm) for the colored products were observed in the UV‒vis spectra (Figure 2b). A series of pH‐, temperature‐ and material concentration‐dependent tests was performed to optimize the catalytic parameters. As shown in Figure 2c, noticeable POD‐like activity was observed for the HEA NPs and compared to the OXD‐like activity, and the optimal pH and temperature were determined to be ≈3.0 and 70 °C, respectively (Figure 2d). Additionally, the oxidation of TMB was also strongly dependent on the concentration of the HEA NPs under the given conditions (Figure 2e). To evaluate the enzymatic activities of the POD mimics, a steady‐state kinetic study was carried out to obtain the rate constants of the HEAs by varying the concentration of H_2_O_2_ with a constant concentration of TMB or vice versa. Typical Michaelis‒Menten curves and Lineweaver‒Burk double reciprocal plots were fitted to obtain the Michaelis constant (*K_m_*) and maximum reaction rate (*V_max_*) of the HEAs, and the calculated parameters are listed in Table S1 (Figure 2f,g; Figures S13 and S14, Supporting Information). As expected, the HEAs with a defined morphology exhibited higher POD‐like activity and improved kinetic parameters compared to aggregated irregular alloys, which was possibly caused by exposure to more active sites on the surface (Figure S15, Supporting Information).^[^ ^19^, ^20^ ^]^ Compared with the nanozymes addressed in previous studies, the HEA NPs exhibited superior affinities and catalytic efficiencies for substrates (*K_m_* = 0.07 and 0.6 mm for TMB and H_2_O_2_; *K_cat_/K_m_* = 1.74 × 10^12^ and 5.38 × 10^11^ for TMB and H_2_O_2_, respectively; Table S2, Supporting Information). These results suggested that HEA NPs had remarkably higher POD‐like activity than HEA bulk (specific activity: 109.65 U mg^−1^ for HEA NPs and 43.48 U mg^−1^ for HEA bulk) 0and exhibited acceptable catalytic stability during long‐term preservation at a wide range of temperatures and pHs (Figure 2h; Figure S16, Supporting Information).

The catalytic mechanism of the HEA POD mimic was investigated with ESR spectroscopy, and strong ESR signals for ^•^OH, ^•^O_2_^−^, and ^1^O_2_ were detected when the HEA NPs were mixed with H_2_O_2_ (Figure 2i). Therefore, the ESR spectra indicated that the HEA NPs catalyzed the decomposition of H_2_O_2_ to produce reactive oxygen species (ROS) such as ^•^OH, ^•^O_2_^−^, and ^1^O_2_, which is consistent with the mechanism hypothesized for metallic POD mimics.^[^ ^39^ ^]^ In contrast, only weak signals of ^•^OH, ^•^O_2_^−^, and ^1^O_2_ were observed in ESR spectra in the absence of H_2_O_2_ (Figure S17, Supporting Information), which suggests that HEA NPs only exhibit limited OXD‐like activity compared to their POD‐like activity. This finding is consistent with the results of the pH optimization.

### 2.3 DFT Calculations for POD‐Like Activity of the HEA NPs

**Figure 3:**
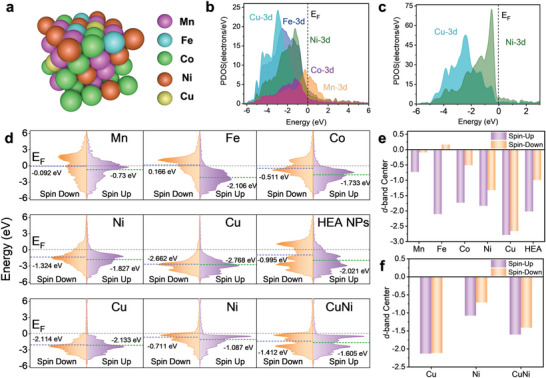
DFT calculation of the electron distribution and structural configuration. a) 3D atomic model showing the crystal structure of the HEA NPs. b,c) PDOSs of the HEA NPs and CuNi, respectively. d) Calculated PDOSs and d‐band centers (including spin‐up and spin‐down) for each element and the bulk HEA NPs and CuNi. e,f) d‐band center comparisons for the individual elements and the bulk HEA NPs and CuNi, respectively.

First‐principles DFT calculations were used to investigate the POD catalytic performance of the HEA NPs. It is well known that the catalytic efficiency of a nanozyme mainly depends on its surface atomic composition and structure, which generally have significant impacts on adsorption or electron transport during catalysis.^[^ ^18^, ^29^ ^]^ Therefore, a lattice model with the FCC random phase for a relatively Fe‐, Ni‐ and Cu‐enriched surface was proposed based on the ICP‒OES, HRTEM, and XRD results (**Figure** **3**a). From the lattice parameters revealed by the XRD and HRTEM characterization, it was inferred that the CuNi bimetallic solid solution phase could well be an integral matrix or constituent phase of the HEA crystal. Also, the abundant Fe sites on the surface of HEA NPs are indispensable for their excellent POD‐like activity. Hence, in this section, the CuNi bimetallic alloy and FeCuNi trimetallic alloys were selected for comparison of the electronic structures and catalytic mechanisms.^[^ ^40^ ^]^ The partial projected density of states (PDOSs) for the HEA NPs, CuNi, and FeCuNi are presented in Figure 3b,c and Figure S18 (Supporting Information) to illustrate their electronic structures. Apparently, the Cu, Fe, and Ni sites were electron‐rich, and HEA exhibited a higher electron abundance at the *E*_F_due to the contributions of Mn, Fe, and Co (Figure S19, Supporting Information). Distinct d‐orbital overlaps among the different metals were clearly observed, demonstrating that the elements in the alloy were strongly bonded to each other. On the other hand, the d‐electron complementation effect amplified the synergistic effect of the d‐electrons from different metal sites. In brief, in the alloy lattice, the internally paired d‐electrons from the late transition metals were likely redistributed into empty or half‐filled vacant d‐orbitals of the early transition metals, resulting in strong d‐orbital coupling.^[^ ^37^, ^41^ ^]^ Thus, the efficiency for site‐to‐site electron transfer of the alloy between the constituent metals was potentially increased, which in turn activated the peroxide substrate with the POD mimics.^[^ ^24^ ^]^ The Cu‐3d band showed a sharp peak at −2.95 eV, which was the furthest position from the *E*_F_, indicating that Cu served as an electron reservoir to maintain the valence balance of the HEsymes during catalysis. It is clear that the Mn‐3d band spanned the E_F_ with high electron density and acted as an electron consumption center during catalysis, making it easier to transfer electrons from the alloy surface to the adsorbed substrate. Additionally, the Fe, Co, and Ni 3d orbitals were located in the middle and exhibited broad bands, both of which contributed to the stabilization of the intermediates and reduction of the energy barrier for electron transfer during the redox reaction.

Figure 3d–f depicts the spin up/down d‐orbitals and d‐band centers of elements in the HEA NPs and CuNi. According to the d‐band center theory and Sabatier principle, a rational upshift of the d‐band center position relative to the E_F_ generally enhances the bond strengths between the metal atoms and oxygen intermediates, thus facilitating the POD‐like activity of the nanozyme.^[^ ^17^, ^42^, ^43^ ^]^ For spin‐polarized transition metal active sites, such as those of Mn, Fe, and Co, the spin‐down d‐orbitals usually occupy higher energy levels relative to the E_F_ due to their lower occupancy levels, and they are more likely to be involved in intermediate adsorption.^[^ ^44^, ^45^ ^]^ Taking into account the delocalization and the strong interactions of the d‐orbital electrons in the alloy, the overall d‐band center was calculated as a descriptor. CuNi showed a low d‐band center (spin‐down, −1.412 eV) owing to the high occupancy of the Cu and Ni 3d orbitals, which normally disfavors the adsorption of oxygenated intermediates.^[^ ^11^, ^46^ ^]^ Compared with CuNi, the contribution of Fe sites results in a noticeable enrichment of d‐electrons near the E_F_ for FeCuNi, which is particularly prominent in the spin‐down electron distribution. As such, the d‐band center of FeCuNi displays a pronounced upshift (−0.885 eV) relative to the *E*_F_ when compared to CuNi (Figures S20 and S21, Supporting Information). Likewise, the spin‐down d‐band center of the HEA bulk was explicitly balanced at a higher position of −0.995 eV with respect to the E_F_ because the Co and Mn 3d orbitals with higher d‐band centers overlapped with the Cu and Ni 3d orbitals. The upshift of the d‐band center relative to the E_F_ described above facilitated the adsorption and stabilization of the oxygen intermediates and may well be considered an index for the reliable catalytic performance of the HEA NPs.^[^ ^17^, ^41^ ^]^ In conclusion, the d‐electron structure of the HEA NPs was regulated by different constituent metals, which guaranteed efficient and stable POD‐like activity.

**Figure 4:**
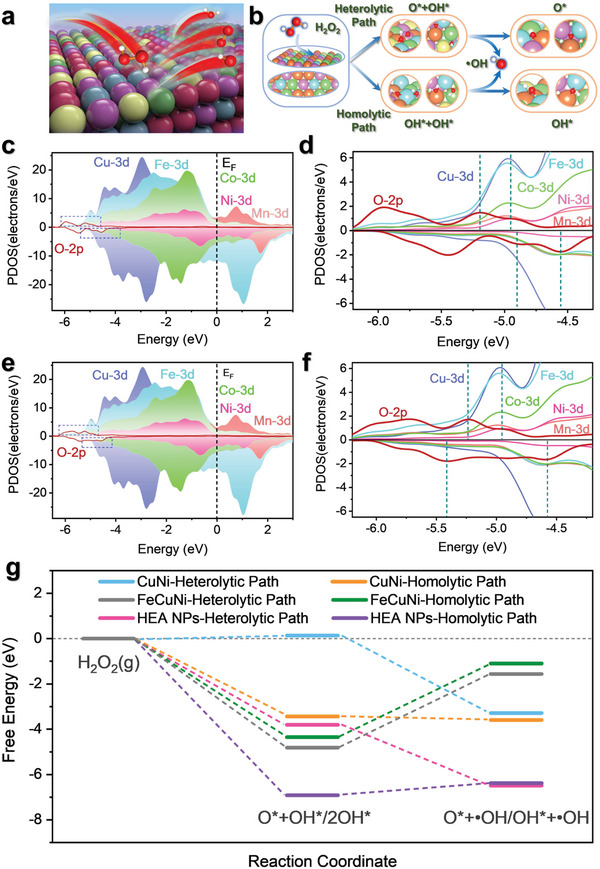
Theoretical calculations of the POD‐like mechanism of the HEA NPs. a) Scheme showing the surface reactivity of the HEA NPs during POD‐like activity. The red and white balls represent O and H, respectively. b) Different reaction pathways for the decomposition of H_2_O_2_ to generate ^•^OH on the HEA NPs with optimization. The white, red, blue, orange, yellow, pink, and green balls represent H, O, Mn, Fe, Co, Ni, and Cu atoms, respectively. c,d) PDOSs and partial enlargement for O* and OH* adsorption on the HEA NPs. e,f) PDOSs and partial magnification of 2OH* adsorption. g) Optimized free energy profiles for H_2_O_2_ decomposition along different pathways on the CuNi, FeCuNi, and HEA NPs.

DFT was also used to study the POD‐like mechanism on the HEA surface, and the reaction model was constructed with the (111) lattice plane of the alloy (**Figure** **4**a). As shown in Figure 4b, there were two main pathways for the degradation of H_2_O_2_ in the POD‐like catalytic mechanism, including the homolytic path and the heterolytic path, and the oxygen intermediates generated by bond‐cleavage of H_2_O_2_ subsequently formed ^•^OH in the acidic medium.^[^ ^47^ ^]^ Detailed calculations indicated that when oxygen intermediates were adsorbed on the HEA surface (Figure 4c–f), the 2p orbitals of O overlapped substantially with the 3d orbitals of the HEA metal atoms and supported stable adsorption. As displayed in Figure 4g, both the homolytic and heterolytic pathways for the HEA exhibited negative adsorption energies for the intermediates (−3.81 eV for OH*+O* and −6.92 eV for 2OH*), indicating that 2 OH* and OH*+O* were adsorbed stably. In contrast, spontaneous adsorption of the intermediate OH*+O* from heterolysis on the CuNi surface was more difficult. Benefiting from the contribution of Fe sites, the intermediates of H_2_O_2_ heterolysis and homolysis tend to adsorb spontaneously on the FeCuNi surface and ^•^OH is more likely to be generated through the heterolytic path. Notably, the two pathways for the HEA showed different free energies for the same steps, and there was a low energy barrier of 0.43 eV for ^•^OH generation from 2OH*, revealing that exothermic heterolysis of the H_2_O_2_ was energetically favored for ^•^OH generation on the HEA. Significantly, homolytic ^•^OH generation proceeded freely on the CuNi, whereas on the HEA, this step would be the rate‐determining step. However, this does not mean that the homolysis of H_2_O_2_ on the CuNi surface was thermodynamically more favorable than that on the HEA surface. In fact, the more negative adsorption energy of 2OH* on the HEA suggested a greater exothermicity, which overcame the lower energy barrier and thus facilitated the desorption of ^•^OH from the surface. Despite the higher d‐band center position of FeCuNi than HEA NPs, the generation of ^•^OH is energetically easier to carry out on the surface of HEA NPs. These results indicate that a moderate upshift of the d‐band center with respect to the E_F_ could improve the catalytic activity of the alloy, but an excessive upshift of the d‐band center with respect to the E_F_ would be counterproductive. This finding aligns with the prediction in the d‐band model for a volcano‐type relationship between the d‐band center position relative to the E_F_ of an alloy and its catalytic activity.^[^ ^17^, ^42^, ^43^, ^48^ ^]^ The optimized adsorption configuration of the key oxygen intermediate on the alloy surface is shown in Figures S22 and S23 (Supporting Information). Furthermore, the insufficient coordination and electron‐donating ability of the abundant transition metal atoms on the surface are thought to contribute to the high intrinsic activity of the HEAs.^[^ ^37^ ^]^ Therefore, the HEzymes exhibited markedly higher rates than CuNi in catalyzing the decomposition of H_2_O_2_ (Figure S15, Supporting Information), which enabled faster conversion of the colorless TMB into blue ox‐TMB. In summary, the DFT results provided detailed information on the electronic structure and catalytic mechanism of the HEA NPs and revealed a synergistic effect among the different elements leading to the high POD‐like activity of the HEA NPs.

### 2.4 Application of the HEzymes in Biosensing and Biomedicine

**Figure 5:**
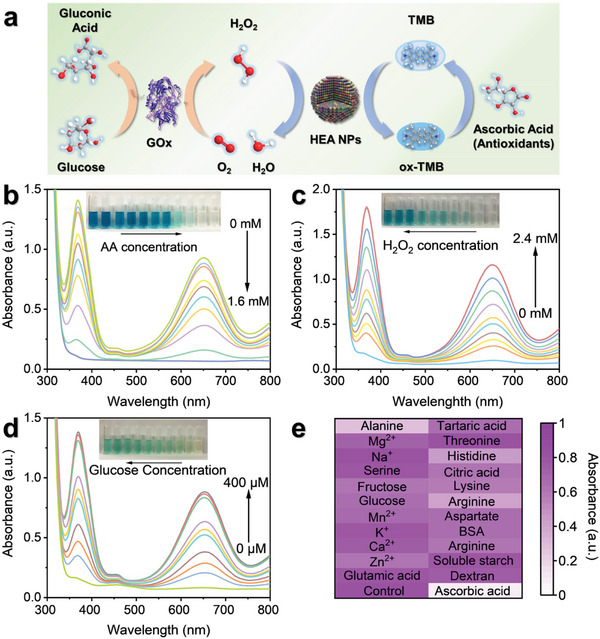
Use of the HEA NPs for Biosensing. a) Mechanism for the proposed detection method based on the POD‐like activity of the HEA NPs (GOX PBD code: 1GAL, [https://www.rcsb.org/structure/1GAL]). b) UV‒vis absorption spectra and visual colors of the HEA/H_2_O_2_/TMB system with different AA concentrations. Incubation conditions: 10 min at room temperature. c) UV‒vis absorption spectra and visual colors of the HEA/TMB system with different H_2_O_2_ concentrations. Incubation conditions: 10 min at room temperature. d) UV‒vis absorption spectra and visual colors of the GOX/HEA/TMB system with different glucose concentrations. Incubation conditions: 15 min at 37 °C. e) Selectivity analysis for the HEA/TMB chromogenic system.

As practical and versatile biosensors, nanozymes are employed in rapid analyses of ascorbic acid (AA), H_2_O_2,_ and glucose (**Figure** **5**a). Food antioxidants, such as AA, polyphenols, and glutathione (GSH), inhibit the oxidation of TMB due to their reducibility.^[^ ^49^ ^]^ Therefore, the HEA/H_2_O_2_/TMB system is sensitive to the antioxidants in the sample, which is reflected by an obvious decrease in the intensity of the absorption peak at 652 nm (Figure 5b; Figure S24a, Supporting Information). As shown in Figure S24b,c (Supporting Information), the ∆absorbance of the HEA/H_2_O_2_/TMB system at 652 nm showed an excellent linear relationship with AA concentration over the range of 40 to 800 µm and a favorable correlation coefficient (*R* ^2^ = 0.9970), and the detection limit of the method was 28.59 µm for the 3S/N (signal/noise) equation. H_2_O_2_ is an essential substrate, and it often serves as a ROS supplier for the chromogenic reactions of POD mimics. On the other hand, GOX catalyzes the oxidation of glucose to produce H_2_O_2_, which can be employed as the substrate for TMB oxidation (Figure 5a).^[^ ^50^ ^]^ As shown in Figure 5c and Figure S25 (Supporting Information), the results demonstrated that the linear range and the LOD (S/N = 3) for the H_2_O_2_ detection assay were 40–400 µm and 15.29 µm, respectively, and the data showed a high correlation coefficient (*R* ^2^ = 0.9970). Likewise, a good linear relationship (*R* ^2^ = 0.9966) between the glucose concentration, ranging from 20 to 160 µm, and the absorbance of the system at 652 nm is depicted in Figure 5d and Figure S26 (Supporting Information), and the LOD (S/N = 3) was 4.00 µm. The effects of potential interferents on the chromogenic reaction were not apparent except for those of alanine, histidine, and arginine, which indicated a satisfactory selectivity for the assay (Figure 5e). Moreover, the established colorimetric platform for the detection of TAC and glucose was applied to beverage samples and vitamin C tablets. The results were essentially consistent with the specifications for the food samples, confirming the practicality of the TAC method (Figure S27 and Table S3, Supporting Information). Assay stability and recycling tests demonstrated the excellent reusability and reliability of the HEsymes (Figure S28, Supporting Information). In conclusion, the HEsymes‐based colorimetric platform is useful for measurements of TAC, H_2_O_2,_ and glucose (Tables S4 and S5, Supporting Information).

**Figure 6:**
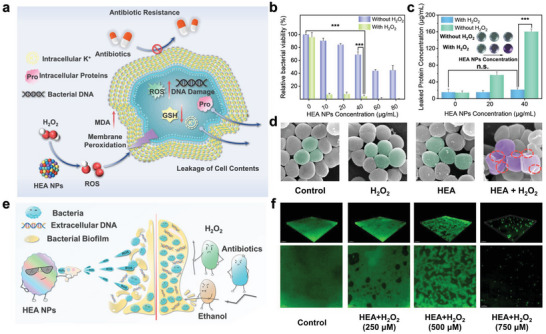
Use of the HEA NPs for Biomedical Applications. a) Principle for ROS‐mediated inactivation of drug‐resistant bacteria based on the HEA NPs. b) Determination of the bactericidal capacity of the HEA/H_2_O_2_ system with the MTT assay. The data are presented as the mean ± SD (*n =* 3 independent samples). c) Leakage of bacterial intracellular proteins. The data are presented as the mean ± SD (*n =* 3 independent samples). d) Typical SEM images of MRSA after different treatments. e) HEA nanozyme‐mediated ROS generation inhibits MRSA biofilms at low H_2_O_2_ concentrations. f) 3D CLSM images of biofilms formed by MRSA with different treatments. One‐way ANOVA and one‐sided Tukey's multiple comparison tests were performed to evaluate the differences in the means of the groups (significance level: **p<0.01, ***p<0.001).

POD mimics are indispensable mediators in recent studies on nanozyme‐based antibacterial and antitumor therapies, whose POD‐like activity exerts a decisive influence on therapeutic efficacy.^[^ ^39^, ^47^, ^51^ ^]^ Dispersion tests revealed that HEA NPs with superior POD‐like activity exhibited favorable dispersibility in common aqueous matrices, suggesting that HEA NPs hold the potential to be utilized for the development of efficient antibacterial therapeutics in physiological conditions (Figure S29, Supporting Information). Hence, HEsymes have been employed for combating four representative pathogenic bacteria (Gram‐negative: *Escherichia coli* O157:H7 (*E. coli* O157:H7), *Salmonella enteritidis* (*S. enteritidis*). Gram‐positive: methicillin‐resistant *Staphylococcus aureus* (MRSA) and *Listeria monocytogenes* (*L. monocytogenes*)) by catalyzing H_2_O_2_ to generate excess ROS and mediate membrane disruption, which is lethal to bacteria (**Figure** **6**a). The antibacterial effect of the HEA/H_2_O_2_ system was evaluated via the minimum inhibitory concentrations (MIC) study and minimum bactericidal concentration (MBC) study.^[^ ^52^, ^53^, ^54^ ^]^ The corresponding results demonstrated that the HEA NPs/H_2_O_2_ system exhibited remarkable broad‐spectrum antibacterial effects against both Gram‐positive and Gram‐negative bacteria, and it was also more potent in killing Gram‐positive bacteria (Figure 6b; Figures S30—S34 and Table S6, Supporting Information). In fact, the Gram‐dependent antibacterial effect of the HEA NPs/H_2_O_2_ system mainly stems from the differences in cell wall composition and structure between Gram‐positive and Gram‐negative bacteria, which is consistent with the trend of several previous findings.^[^ ^51^, ^55^, ^56^, ^57^ ^]^ To further investigate the bactericidal mechanism of the HEA NPs/H_2_O_2_ antibacterial system, MRSA was used as a model strain for the measurement of biochemical indicators after different treatments. The live/dead bacterial staining reveals that the HEA/H_2_O_2_ groups exhibit the most effective synergistic bactericidal effect (Figure S35, Supporting Information). The membrane permeability test and the MDA assay demonstrated that bacterial death caused by the HEA/H_2_O_2_ system arose from peroxidative damage to the bacterial membrane and was correlated with the concentration of the HEA NPs (Figure 6c; Figure S36a,b, Supporting Information). Meanwhile, the virulence of MRSA, such as the hemolytic capacity and plasma coagulase activity, explicitly declined after the treatment with HEA/H_2_O_2_, which was mainly due to the death of MRSA organisms caused by ROS attack (Figure S36c,d, Supporting Information). The GSH depletion capacity of the HEA/H_2_O_2_ system disrupted the antioxidant system of the bacteria, thereby enhancing the bactericidal capacity (Figure S37, Supporting Information). DCFH‐DA staining demonstrated that both the dramatic elevation of bacterial endogenous ROS levels and the membrane damage mediated by ROS outbreaks were closely correlated with the bactericidal mechanism of the POD mimetic enzymes (Figure S38, Supporting Information). The morphologies of the bacteria after the application of different therapies are displayed in Figure 6d. In contrast to the control group, the attack of ROS for the HEA/H_2_O_2_ group caused nonnegligible wrinkling and destruction of the bacterial surface. As a consequence, irreversible damage and changes in the permeabilities of the bacterial membranes occurred, leading to leakage of the endogenous contents and triggering bacterial death. The biocompatibility evaluation suggested that the HEA NPs exhibited desirable hemocompatibility and cytocompatibility, which can be used for the elimination of drug‐resistant bacteria (Figure S39 and Table S7, Supporting Information).

Multidrug‐resistant bacterial infections, especially those accompanied by biofilm formation, present considerable challenges for wound therapy.^[^ ^58^ ^]^ The formation of multidrug‐resistant bacterial biofilms inhibits the permeation of antibiotics and conventional bactericides and facilitates the horizontal transfer of pathogenic and drug‐resistance genes among bacteria.^[^ ^59^ ^]^ In particular, extracellular DNA (eDNA) is an integral part of the bacterial biofilm matrix, and it is capable of cross‐linking with extracellular polysaccharides to create a “shelter” for the bacteria.^[^ ^59^, ^60^ ^]^ Herein, the HEA NPs with POD‐like activity, acted as protagonists and used H_2_O_2_ as a weapon to provoke an outbreak of ROS that damaged the organism and lysed the biofilm (Figure 6e). As shown in Figure 6f and Figures S40 and S41 (Supporting Information), due to the synergy of the HEA NPs and H_2_O_2_, MRSA biofilm formation was significantly inhibited, and the concentration of eDNA in the biofilm was remarkably lower than that in the control group. These results indicated that ROS burst‐mediated nanocatalysis could be adopted as an efficient and safe countermeasure to biofilm formation by drug‐resistant bacteria.

## 3 Conclusion

We have developed a class of novel HEzymes with multiple tailored active sites and tuned electronic structures. The strong electron interactions among the constituent metals enhanced the catalytic efficiency, as manifested by the regulated electron distribution of the bulk near the E_F_ and the rational shift of the d‐band center of the bulk relative to the E_F_. With a POD nanocatalyst as a model, detailed analyses indicated that the proposed HEA POD mimic with an FCC crystal structure exhibited remarkable binding affinity and catalytic efficiency comparable to those of natural enzymes. The d‐electron complementary effect and synergy endowed the HEzymes with significantly higher catalytic efficiencies than conventional nanozymes. Moreover, HEzymes exhibited desirable performance in target compound detection, drug‐resistant bacteria elimination, and bacterial biofilm eradication. In general, this work is intended to provide an original paradigm for the exploitation of high‐performance nanozymes with enhanced catalytic activities and to make HEAs a resource‐rich and versatile platform for the effective design and application of nanozymes.

## 4 Experimental Section

### Reagents

Manganese (II) acetylacetonate (Mn(acac)_2_), cyltrimethylammonium bromide (CTAB), stearyl trimethyl ammoium bromide (STAB), Triton X‐100 and 2,2′‐Azino‐bis (3‐ethylbenzothiazoline‐6‐sulfonic acid) diammonium salt (ABTS) were purchased from Shanghai Sigma‐Aldrich Trading Co., Ltd. (Shanghai, China). Iron (III) acetylacetonate (Fe(acac)_3_), oleylamine (OAm), fluorescein diacetate (FDA), and molybdenum hexacarbonyl Mo(CO)_6_ were acquired from Macklin Biochemical Technology Co., Ltd (Shanghai, China). Cobalt acetylacetonate (III) (Co(acac)_3_), nickel (II) acetylacetonate (Ni(acac)_2_), copper (II) acetylacetonate (Cu(acac)_2_), 3,3′,5,5′‐tetramethylbenzidine (TMB), o‐phenylenediamine (OPD), l‐glutathione reduced (GSH), 5,5′‐dithio bis‐(2‐nitrobenzoic acid) (DTNB), 2′7′‐dichlorodihydrofluorescein diacetate (DCFH‐DA) and NaBr (FT‐IR grade) were purchased from Shanghai Aladdin Biochemical Technology (Shanghai, China). Glucose, dimethyl sulfoxide (DMSO, C_2_H_6_OS), ethanol (CH_3_CH_2_OH), acetic acid (CH_3_COOH), sodium acetate trihydrate (CH_3_COONa), H_2_O_2_ (30%), and ascorbic acid were obtained from Guangdong Guanghua Technology Co., Ltd. (Guangdong, China). 4′,6′ ‐diamidino‐2‐phenylindole (DAPI), propidium iodide (PI), 3‐(4,5‐dimethylthiazol‐2‐yl)−2,5‐diphenyltetrazolium bromide (MTT), glutaraldehyde (2.5% (EM Grade)), sterile defibrinated sheep blood and crystal violet (CV) were purchased from Beijing Solarbio Science & Technology Co., Ltd (Beijing, China). Ezup Column Bacteria Genomic DNA Purification Kit was obtained from Tiangen Biochemical Technology Co., Ltd (Beijing, China). Protein quantification kit (BCA Assay), micro reduced glutathione (GSH) assay kit, and micro lipid peroxidation (MDA) assay kit were obtained from Abbkine Scientific (Wuhan, China). Luria‐Bertani (LB) broth, tryptone soy broth (TSB), LB nutrient agar, and freeze‐dried rabbit plasma were purchased from Beijing Land Bridge Technology Co., Ltd (Beijing, China). Other reagents were all obtained locally. All experimental aqueous solutions were prepared with distilled water.

### Synthesis of MnFeCoNiCu HEA NPs

MnFeCoNiCu HEA NPs were synthesized according to previous reports with a slight modification.^[^ ^23^, ^33^, ^37^ ^]^ CTAB (90 mg) was added into a flask containing OAm (5 mL). After ultrasonication for 30 min, Mn(acac)_2_ (31 mg), Fe(acac)_3_ (42 mg), Co(acac)_3_ (9 mg), Ni(acac)_2_ (9 mg) and Cu(acac)_2_ (10 mg) were successively added. Then the mixture was sonicated with glucose for 2 h to obtain a homogeneous solution. After that, nitrogen was injected into the flask and the solution was heated to 220 °C for 6 h under magnetic stirring in an oil bath. The mixture was rapidly cooled to room temperature and the black products were collected by centrifugation and washed three times with an ethanol/cyclohexane mixture (v/v: 9:1). Finally, the precipitates were lyophilized and dispersed in distilled water for further experiments. In addition, other reference materials in the work were synthesized under the same conditions and processes as the proposed approach, except for the different formulations of the ingredients.

### Characterization of MnFeCoNiCu HEA NPs

TEM and HRTEM images were characterized by a JEM 2100F (JEOL, Japan) at an accelerating voltage of 200 kV. EDS was obtained by PV97‐617300‐ME (AMETEK, US). Powder XRD patterns were recorded on an X‐ray diffractometer (D8 Advance, Bruker, Germany). XPS were obtained by an Axis Ultra DLD instrument (Kratos Analytical, UK) equipped with an Al Kα X‐ray source (1486.6 eV). ICP‐OES (720ES, Agilent, US) was used to determine the compositions of HEA NPs. FT‐IR spectra were obtained on the wavenumber range of 400—4000 cm^−1^ with a Vetex70 (Bruker Corp, Germany) instrument using the KBr pellet method. All UV‐visible absorption spectra were recorded using a UV–vis spectrophotometer (UV‐2550, Shimadzu, Japan). A microplate reader (Multiskan MK3, Thermo Fisher Scientific, US) was used to measure the absorption at 652 nm of all test groups.

The configurational entropy of the high‐entropy alloy was defined by the following equation:

(1) $$\Delta S_{\mathrm{configuration}}=-R\sum_{i=1}^{n}x_{i}\ln x_{i}$$

(∆*S*_configuration_ >1.5 R: high‐entropy; 1.0–1.5R middle (or medium)‐ entropy; <1.0R low‐entropy class.)

In this work, ∆*S*_configuration_ for MnFeCoNiCu NPs = 1.52 R, so HEA NPs are classified as high‐entropy materials.

### Enzyme‐Like Activity of MnFeCoNiCu HEA NPs

The OXD‐ and POD‐like activity of HEA NPs were determined by TMB colorimetric assays.^[^ ^61^ ^]^ In a typical test, TMB (1 mm) and H_2_O_2_ (10 mm) were added into pH 3.0 HAc‐NaAc buffer (0.1 m) containing 6 µg mL^−1^ HEA NPs and absorbances of the mixtures at 652 nm were recorded at room temperature (reaction time = 10 min). Meanwhile, the OXD‐like activity of HEA NPs was determined without the addition of H_2_O_2_.

To explore the optimal conditions of the enzyme‐like activity of HEA NPs, a range of pH values (2.0 to 7.0) and temperature (20 to 80 °C) for the reaction were tested to investigate the oxidation of TMB. Moreover, the time‐dependent kinetics were also conducted to optimize the incubation time and enzyme concentration of the reaction.

### Enzyme Kinetics Studies of MnFeCoNiCu HEA NPs

A steady‐state kinetics assay of HEA NPs was performed in pH 3.0 HAc‐NaAc buffer (0.1m) by changing the concentrations of H_2_O_2_ (5.0 to 70 mm) with a fixed concentration of TMB (1.0 mm) or changing the concentrations of TMB (0.1 to 1.0 mm) while maintaining the concentration of H_2_O_2_ (20 mm). The Michaelis‐Menten constant was calculated using Lineweaver‐Burk plots of the double reciprocal of the Michaelis‐Menten equation *v* = *v_max_* ×[S]/(*K_m_* +[S]), where v is the initial velocity, *v_max_* is the maximal reaction velocity, [*S*] is the concentration of substrate, and the *K_m_* is the Michaelis constant.

### Determination of Specific Activity

Briefly, a series of concentrations of HEA NPs were mixed with H_2_O_2_ (1 m) and TMB (0.5 mg mL^−1^) in 2 mL HAc‐NaAc buffer (pH 3.0, 0.1 m), and the initial reaction rate of the solution was calculated from the absorbance variations of TMB (∆*A*/∆*t*). A linear fit curve of the nanozyme concentration to the initial rate of reaction was performed to determine its specific activity.

### ESR Measurement

An Electron spin‐resonance (ESR) spectroscopy spectrometer was utilized to measure the generation of hydroxyl radicals (^•^OH), superoxide anion (^•^O_2‐_), and singlet oxygen (^1^O_2_). Briefly, the tests were conducted under the following conditions including H_2_O_2_, DMPO, and HEA NPs. All mixtures were dispersed in pH 3.0 HAc‐NaAc buffer (0.1 m). The solutions were subjected to ESR analysis immediately after reacting for 10 min at room temperature.

### Colorimetric Detection of Ascorbic Acid

Ascorbic acid was used as a representative antioxidant to establish an indirect method for the total antioxidant capacity of samples. HEA NPs (6 µg mL^−1^) and H_2_O_2_ (20 mm) were added into pH 3.0 HAc‐NaAc buffer (0.1 m). Then, TMB (1.0 mm) with varying concentrations of ascorbic acid was added to the mixture. The absorption spectra of the systems were measured after incubation for 10 min at room temperature.

### TAC Detection in Food Samples

Several commercial beverages and two vitamin C tablets were preprocessed to fit the determination range of the AA detection assay, which included dissolution and dilution. After that, the same protocol for AA detection was applied to the TAC detection of samples. Therefore, the TAC of two vitamin C tablets was evaluated to validate the accuracy and feasibility of the AA detection assay mentioned above.

### Colorimetric Detection of H_2_O_2_

HEA NPs (6 µg mL^−1^) and TMB (1.0 mm) were added into pH 3.0 HAc‐NaAc buffer (0.1 m) containing varying concentrations of H_2_O_2_. The absorption of the determination system at 652 nm was recorded after incubation for 10 min.

### Colorimetric Detection of Glucose

HAc‐NaAc buffer (pH 3.0, 0.1 m) containing GOX (1.0 mg mL^−1^), HEA NPs (6 µg mL^−1^) TMB (1.0 mm), and glucose with various concentrations was incubated for 15 min at 37 °C. Subsequently, the absorption of the solution at 652 nm was recorded at room temperature. The protocol above was also applied to the glucose detection of food samples. Above all, the concentration of all samples was adjusted to the linear range of the glucose detection assay.

### Selectivity Test of the Detection Assay

To assess the selectivity for AA, H_2_O_2,_ and glucose in the detection assay, certain substances that commonly occur in food samples were added into the detection system, with concentrations of these interference factors or times that of AA. The absorbances of all test groups at 652 nm were recorded at room temperature after incubation for 10 min.

### Preparation of Bacterial Suspensions

Four representative pathogenic bacteria (Gram‐negative: *Escherichia coli* O157:H7 (*E. coli* O157:H7), *Salmonella enteritidis* (*S. enteritidis*); Gram‐positive: methicillin‐resistant *Staphylococcus aureus* (MRSA) and *Listeria monocytogenes* (*L. monocytogenes*)) were chosen as model bacterial strains. The bacteria were cultured on an LB agar plate at 37 °C for 24 h. Afterward, single colonies of bacteria were transferred to 30 mL of liquid LB broth and shaken (180 rpm) for 12 h at 37 °C. The bacteria were harvested by centrifugation (6000 rpm, 3 min, 4 °C) and washed with sterile saline three times. Lastly, the supernatant was discarded, and the bacteria were resuspended in PBS. The concentration of the bacterial suspension was diluted to 10^7^ CFU mL^−1^ for use.

### The Minimum Inhibitory Concentrations (MIC) Study In Vitro

The MIC study for HEA NPs/H_2_O_2_ antibacterial system against MRSA was determined using the broth dilution method. 100 µL of prepared bacterial suspension containing HEA NPs (0 to 80 µg mL^−1^) and H_2_O_2_ (200 µm) or only HEA NPs was transferred into 100 µL of liquid LB broth in a 96‐well plate, and only H_2_O_2_ or MRSA suspension was set as the control. After 24 h of co‐incubation at 37 °C and PBS washing, the OD_600_ values of the bacterial suspensions were recorded.

### MTT Assay for In Vitro Antibacterial Study

The bacterial organisms from the MIC study were centrifuged (6000 rpm, 3 min) and resuspended in 100 µL PBS. Then, MTT (0.5 mg mL^−1^, filtered with 0.22 µm nylon) was added to each group. After the incubation (37 °C, 4 h), the precipitate was collected by centrifugation (6000 rpm, 3 min), and 100 µL DMSO was added. Finally, the absorbance of each tube was recorded at 570 nm.^[^ ^62^, ^63^, ^64^ ^]^

### The Minimum Bactericidal Concentrations (MBC) Study In Vitro

Based on the results of MIC studies, multiple HEA NPs dose‐increasing HEA/H_2_O_2_ groups were set up near the MIC of the HEA/H_2_O_2_ system against each strain. Briefly, 100 µL of prepared bacterial suspension containing HEA NPs (45 to 60 µg mL^−1^ and H_2_O_2_ (200 µm) or only HEA NPs was transferred into 100 µL of liquid LB broth and co‐incubated at 37 °C for 24 h. The bacteria in each group were collected by centrifugation and resuspended in 100 µL of PBS buffer. The OD_600_ values of the bacterial suspensions after different treatments were recorded. Subsequently, 0.5 mg mL^−1^ of MTT was added to the system and incubated for 4 h at 37 °C. The system was centrifuged (6000 rpm, 3 min) and the supernatant was discarded. The resulting formazan was dissolved in 100 µL DMSO and the absorbance of the solution at 570 nm was determined.

### Bacterial Membrane Permeability Test

The prepared MRSA suspensions were incubated with different groups: (I) MRSA suspensions, (II) HEA NPs (40 µg mL^−1^), (III) H_2_O_2_ (200 µm), (IV) HEA NPs + H_2_O_2_. After incubation at room temperature for 2 h, the bacterial suspensions after different treatments were collected for use.

The quantitative determination of protein leakage in each sample was conducted by a protein quantification kit (BCA Assay). Intracellular K^+^ leakage from bacteria was measured by an atomic absorption spectrophotometer (ZEEnit 700P, Germany).

### GSH Consumption Capacity of HEA NPs

Ellman's assay was conducted to measure the GSH consumption properties of HEA NPs. Various concentrations of HEA NPs were mixed thoroughly with GSH (0.5 mm) in Tris‐HCl buffer (0.05 m, pH 8.0). Then, the absorbance‐time curves of all mixtures were recorded after the addition of DTNB (0.1 mm) and H_2_O_2_.

### Estimation of Bacterial Membrane Peroxidation Levels and Loss of GSH

The level of peroxidation of bacterial cell membranes can be indicated by the content of MDA. Bacterial organisms after different treatments were obtained by centrifugation (8000 rpm, 5 min). Subsequently, The GSH level and the MDA level were measured by following the instructions of the micro‐reduced glutathione assay kit and micro lipid peroxidation assay kit.

### Live–Dead Staining and Detection of Intracellular ROS

After different processing, MRSA suspensions were stained with DAPI/PI (5 µg mL^−1^, 500 µg mL^−1^) and DCFH‐DA (50 µm) at 37 °C for 2 h, respectively. The solution was centrifuged three times and resuspended in PBS to remove excess dye. All samples were observed and analyzed using a confocal laser scanning microscopy (CLSM, TCS SP8, LEICA, Germany). All operations have to be performed under light‐proof conditions.

### Bacteria Morphology Observation

Different groups of treated bacterial suspensions were washed with sterile saline. The sterile glass slices were immersed in different samples for 15 min. Then, the samples were centrifuged (8000 rpm, 5 min) and fixed with 2.5% glutaraldehyde for 8 h. After dehydration with increasing concentrations of aqueous ethanol (30%, 50%, 70%, 80%, 90%, 100%) for 10 min each, the samples were immersed in isoamyl acetate. Finally, the samples were dried by supercritical fluid desiccation and observed with a scanning electron microscope (SEM, S‐4800 FE‐SEM, Hitachi, Japan).

### Bacterial Virulence Test

Briefly, 500 µL of sterile saline was injected into a penicillin vial containing lyophilized rabbit plasma. Then, 300 µL of the treated bacterial suspension collected in each antibacterial activity test group was injected into the prepared rabbit plasma and mixed well. The penicillin vials were incubated at 37 °C, and the results were observed within 6 h. Similarly, the different treated bacterial suspensions were mixed with red blood cells washed with PBS (pH 7.4) and co‐incubated at 37 °C for 5 h. Finally, the samples were centrifuged (2500 rpm, 10 min), and the supernatant was retained and its absorbance was measured at 540 nm.

### Hemolytic Test

The sterile defibrinated sheep blood was centrifuged (2500 rpm, 10 min) to collect the red blood cells. Afterward, the red blood cells were washed and resuspended with PBS (pH 7.4). The red blood cell suspensions were added to a 96‐well plate and incubated with different concentrations of HEA NPs. 0.1% Triton X‐100 was used as the positive control and PBS as the negative control. All groups were incubated at 37 °C for 4 h. Then, the plate was centrifuged (2500 rpm, 10 min), and the supernatant of each well was taken to measure its absorbance at 540 nm.

### Cytotoxicity Measurement of HEA NPs

Briefly, after resuscitation, 3T3 cells were transferred into 96‐well plates and co‐incubated with various concentrations of HEA NPs at 37 °C and 5% CO_2_ for 4 h. Subsequently, MTT (5.0 mg mL^−1^) solution was added to each well and incubated for 4 h. DMSO was used to dissolve the produced formazan and the absorbance of the solution was measured at 570 nm.

### Bacterial Biofilm Formation Inhibition Activity

To simulate the initial formation of biofilm under physiological conditions, 1 mL MRSA suspension was fully mixed with 1 mL LB broth (containing 1% glucose), and the mixture was subsequently added dropwise to the surface of sterile cell culture dishes and cultured at 37°C overnight. Then, HEA NPs (40 µg mL^−1^) and H_2_O_2_ of different concentrations (250, 500, and 750 µm) were added, and all samples were incubated at 37 °C for 24 h. PBS was then used to thoroughly rinse off the suspended residues. The formed biofilms were stained with FDA (200 µg mL^−1^) and the excess dye was washed with PBS. The results were analyzed by a spinning disk confocal microscope (Revolution WD, Andor, UK).

### Cv Staining For Assessment of the Biomass of Bacterial Biofilms

First, 500 µL of the prepared MRSA suspension was added into 500 µL liquid LB broth in a 24‐well plate and incubated at 37 °C for 12 h. After that, different test groups were conducted in the wells: (II) HEA NPs (40 µg mL^−1^), (III) H_2_O_2_ (750 µm), (IV) HEA NPs + H_2_O_2_. After incubation at 37 °C for 24 h, the plate was washed with PBS three times, and 400 µL of methanol was added to immobilize the bacterial biofilm for 20 min. Next, 400 µL of 0.1% CV solution was added to stain the biofilm for 15 min. The redundant dye was gently washed with PBS, and then a 33% acetic acid aqueous solution was added after the water was completely dry. Lastly, the absorbance of each well at 595 nm was measured.

### Inhibition of Extracellular DNA in Bacterial Biofilms

The same procedure used in CV staining was employed to form MRSA biofilms of different treatments. Then, the bacterial extracellular DNA inside the biofilm was extracted following the instructions of the Ezup Column Bacteria Genomic DNA Purification Kit without wall‐breaking treatment of the bacterial cells. Ultimately, the extracellular DNA of the bacterial biofilm was determined by a Nano‐200 (Aosheng Instrument Co., Ltd., China), and guaranteed that the ratio of *A*_260_/*A*_280_ was ≈1.8 while the ratio of *A*_260_/*A*_230_ was located at 2.0‐2.2.

### Density Functional Theory Computational Method

In this work, the Vienna Ab Initio Package (VASP)^[^ ^65^, ^66^ ^]^ was employed to perform all the density functional theory (DFT) calculations within the generalized gradient approximation (GGA) using the PBE^[^ ^67^ ^]^ formulation. The projected augmented wave (PAW) potentials^[^ ^68^, ^69^ ^]^ were chosen to describe the ionic cores and take valence electrons into account using a plane wave basis set with a kinetic energy cutoff of 400 eV. Partial occupancies of the Kohn−Sham orbitals were allowed using the Gaussian smearing method and a width of 0.05 eV. The electronic energy was considered self‐consistent when the energy change was smaller than 10^−5^ eV. A geometry optimization was considered convergent when the force change was <0.02 eV Å^−1^. Grimme's DFT‐D3 methodology^[^ ^70^ ^]^ was used to describe the dispersion interactions.

Based on the results of previous experiments, the equilibrium lattice constant of FCC (face‐centered cubic) Mn_0.125_Fe_0.313_Co_0.083_Ni_0.229_Cu_0.250_ unit cell was optimized to be *a* = 3.4884 Å. It was then used to construct a (111) surface model with *p*(3×2√3) periodicity in the *x* and y directions and four atomic layers in the *z*‐direction separated by a vacuum layer in the depth of 15 Å in order to separate the surface slab from its periodic duplicates. During structural optimizations, a 4 × 3 × 1 *k*‐point grid in the Brillouin zone was used for *k*‐point sampling, and the bottom two atomic layers were fixed while the top two were allowed to relax.

The free energy of a gas phase molecule or an adsorbate on the surface was calculated by the equation *G = E* + *ZPE* − *TS*, where *E* is the total energy, *ZPE* is the zero‐point energy, *T* is the temperature in kelvin (298.15 K is set here), and *S* is the entropy.

### Statistical Analysis

All data were obtained from at least three independent experiments and presented as mean ± standard deviation (SD). The sample size for each analysis was indicated in the corresponding figure caption. The Student's t‐test was used to evaluate the difference between the means of the two groups (significance level: *p<0.05, **p<0.01, ***p<0.001). The one‐way ANOVA and one‐sided Tukey's multiple comparison tests were performed to evaluate the differences among the means of three or more groups (significance level: *p<0.05, **p<0.01, ***p<0.001). All statistical analyses in this work were performed using Minitab 18.0 software.

## Conflict of Interest

The authors declare no conflict of interest.

## Supporting information

Supporting Information

## Data Availability

The data that support the findings of this study are available from the corresponding author upon reasonable request.
