# Supplementary material for: Transition Metal High‐Entropy Nanozyme: Multi‐Site Orbital Coupling Modulated High‐Efficiency Peroxidase Mimics
Source: Adv Sci (Weinh). 2023 Oct 23;10(33):2303078. doi: 10.1002/advs.202303078 (PMC10667809; doi:10.1002/advs.202303078)
Supplement: Supplementary file 1 — Supporting Information [file ADVS-10-2303078-s001.pdf]

ADVANCED SCIENCE
Open Access

Supporting Information

for *Adv. Sci.*, DOI 10.1002/advs.202303078

Transition Metal High-Entropy Nanozyme: Multi-Site Orbital Coupling Modulated High-Efficiency Peroxidase Mimics

Jianxing Feng, Xuewei Yang, Ting Du, Liang Zhang, Pengfei Zhang, Junchen Zhuo, Linpin Luo, Hao Sun, Yaru Han, Lizhi Liu, Yizhong Shen, Jianlong Wang* and Wentao Zhang**

Supporting Information

Transition metal high-entropy nanozyme: multi-site orbital coupling modulated high-efficiency peroxidase mimics

Jianxing Feng[1], Xuewei Yang[1], Ting Du[1], Liang Zhang[1], Pengfei Zhang[1], Junchen Zhuo[1], Linpin Luo[1], Hao Sun[1], Yaru Han[2], Lizhi Liu[3], Yizhong Shen[4], Jianlong Wang*[1], Wentao Zhang*[1]*

[1]College of Food Science and Engineering, Northwest A&F University, 22 Xinong Road, Yangling 712100, Shaanxi, China. [2]Department of Chemical Engineering, Columbia University, New York, NY 10027, USA. [3]Department of Anesthesiology, Division of Critical Care Medicine, Boston Children's Hospital, Harvard Medical School, Boston, MA 02115, USA. [4]School of Food & Biological Engineering, Key Laboratory for Agricultural Products Processing of Anhui Province, Hefei University of Technology, Hefei 230009, China.

*Corresponding author.

E-mail: zhangwt@nwsuaf.edu.cn, wanglong79@nwsuaf.edu.cn, yzshen@hfut.edu.cn

Fax: +86 29-8709-2275;

Tel: +86 29-8709-2275

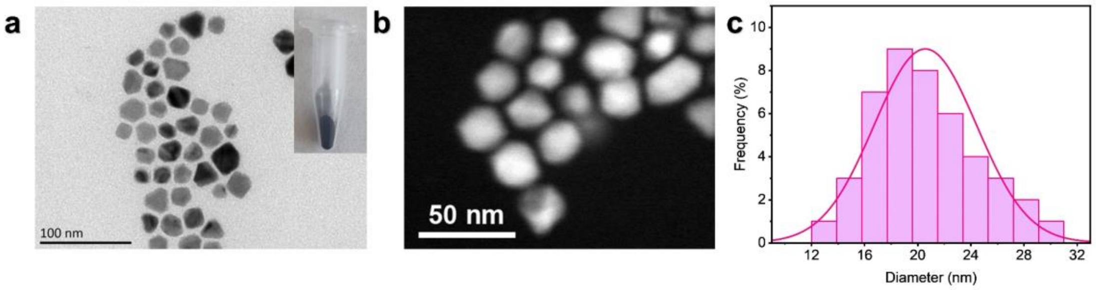

Figure S1. Investigation on the morphology of HEANPs. (a) TEM image and optical photograph (inset image) of MnFeCoNiCu HEA NPs. (b) HAADF-STEM image of HEA NPs. c Size distribution of HEA NPs.

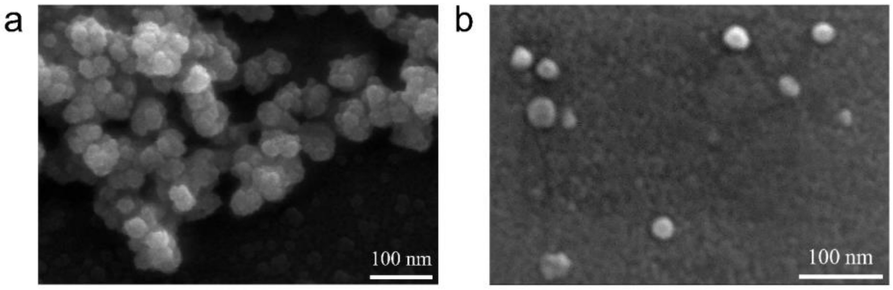

Figure S2. (a, b) SEM images of HEA NPs.

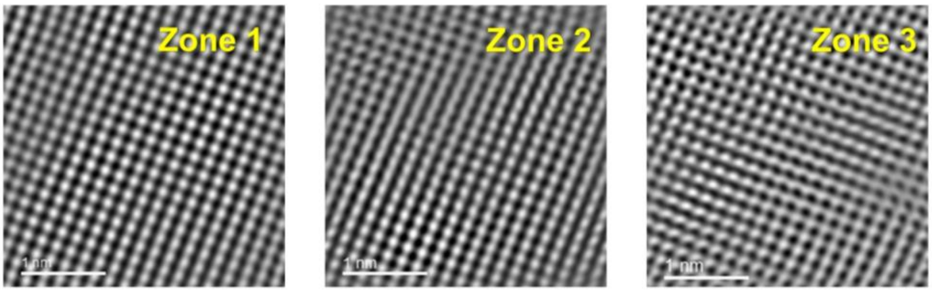

Figure S3. Corresponding IFFT patterns of the Zones in Fig C_1-C_3.

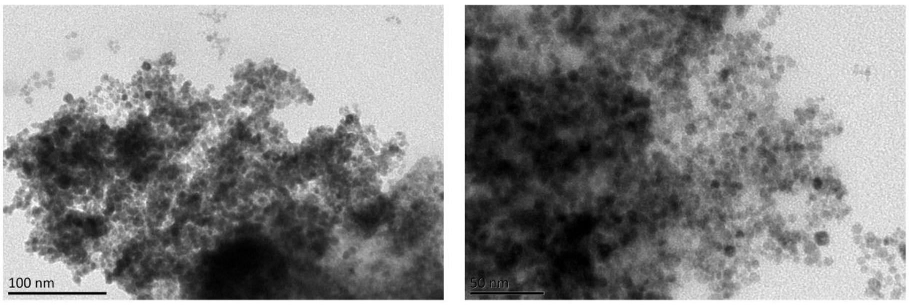

Figure S4. TEM image of the products synthesized using only metal precursors.

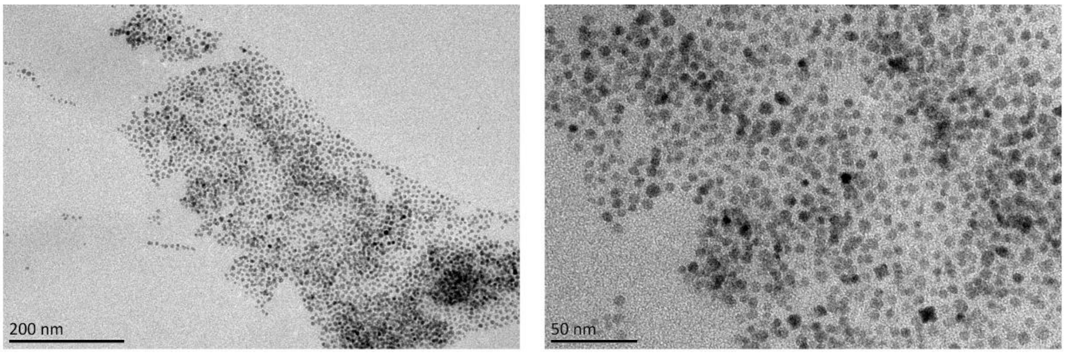

Figure S5. TEM image of the products synthesized using metal precursors and CTAB.

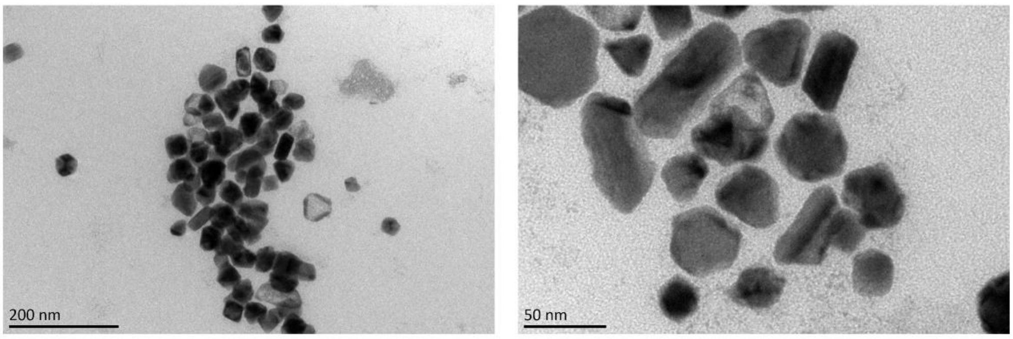

Figure S6. TEM image of the products synthesized using metal precursors, STAB, and glucose.

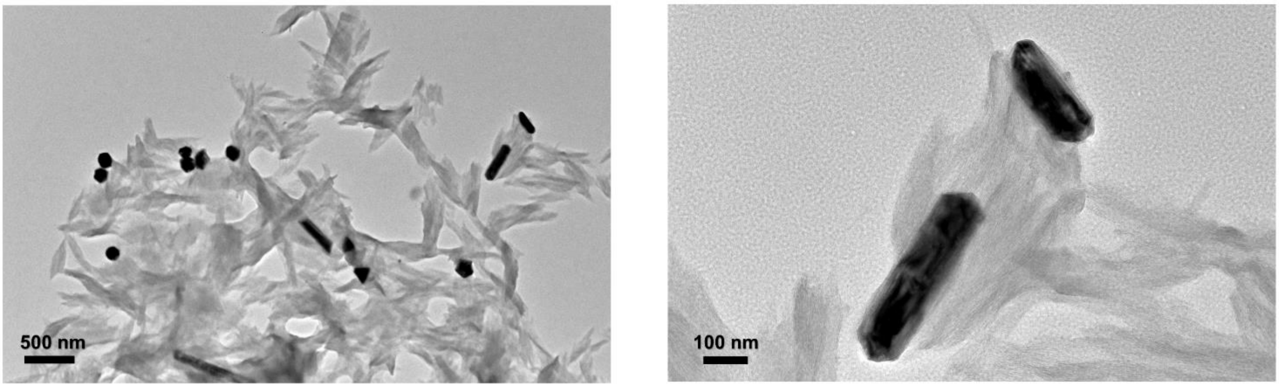

Figure S7. TEM image of the products synthesized using metal precursors, CTAB, glucose, and $Mo(CO)_6$.

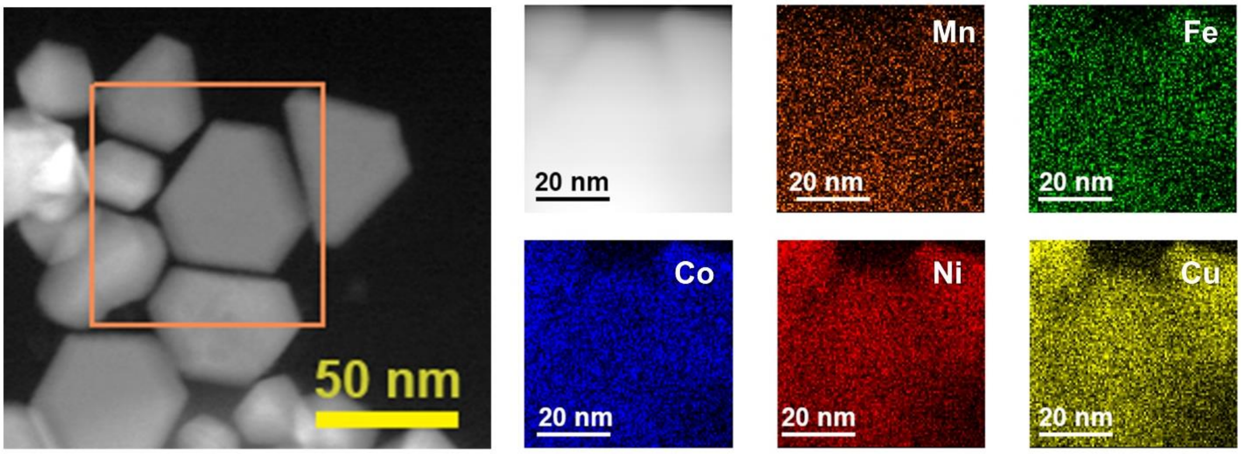

Figure S8. EDS mapping image of HEA NPs.

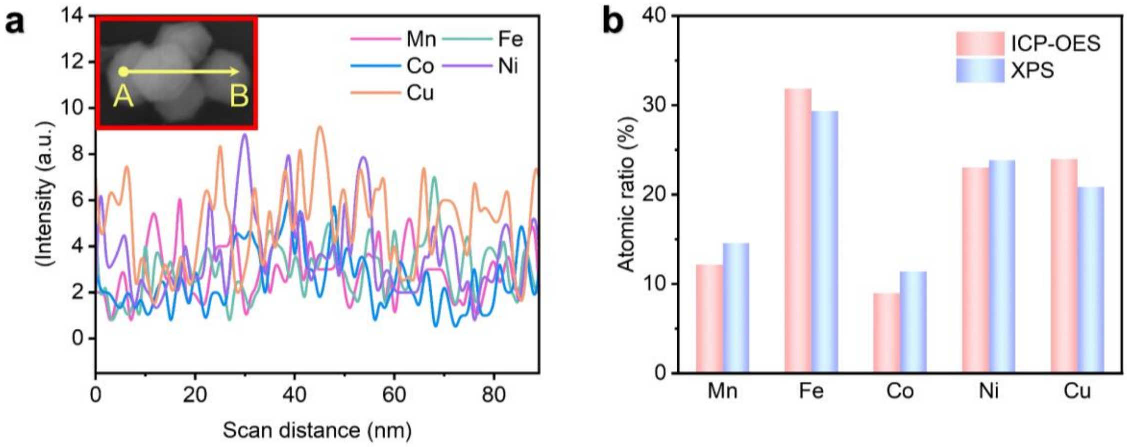

Figure S9. Component element analysis of HEA NPs. (a) The EDS line-scan of HEA NPs. (b) Metal atom ratios of HEA NPs obtained by XPS and ICP-OES results.

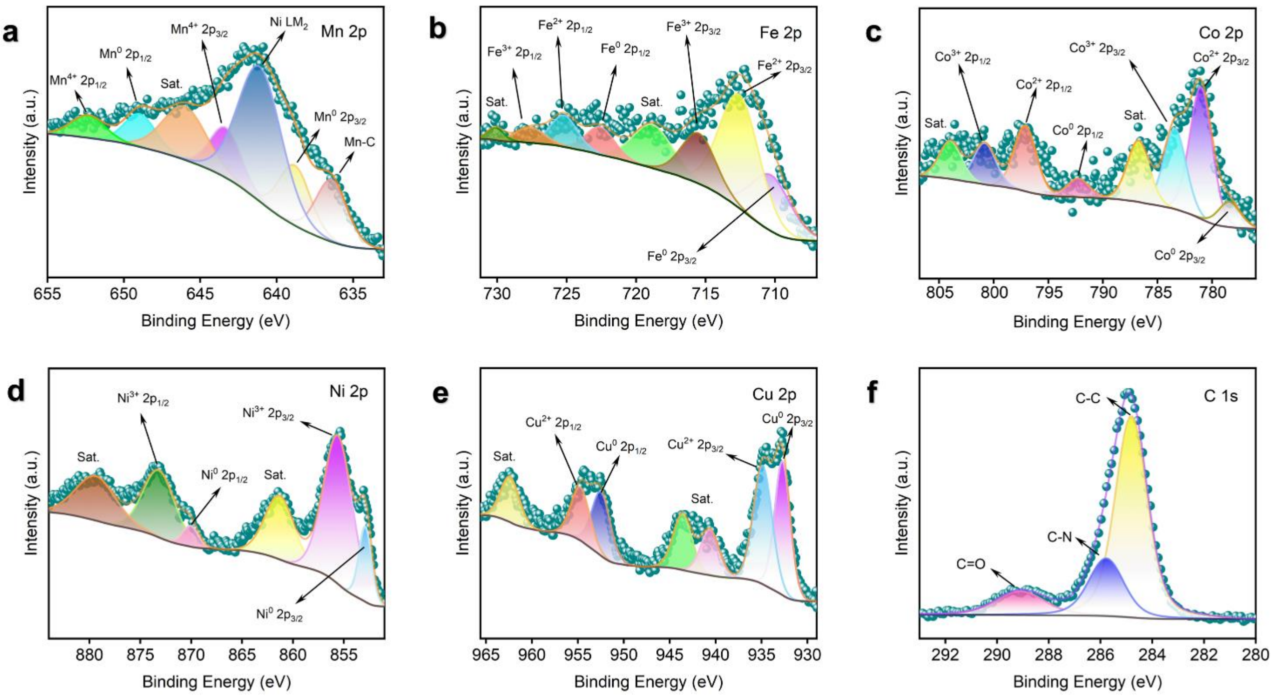

Figure S10. XPS spectra of (a) Mn 2p, (b) Fe 2p, (c) Co 2p, (d) Ni 2p, (e) Cu 2p, and (f) C 1s for HEA NPs.

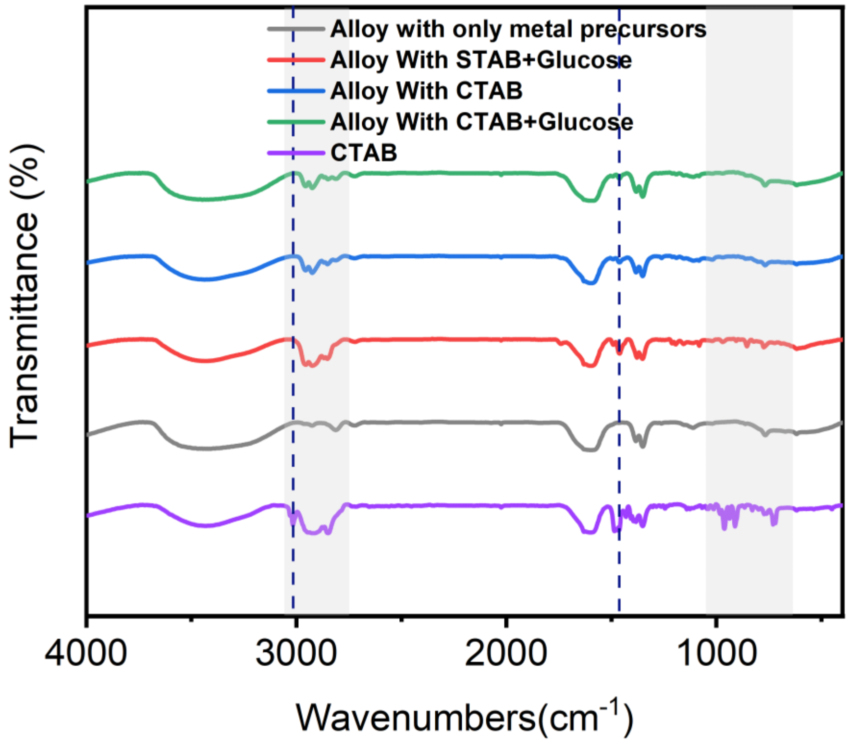

Figure S11. FT-IR spectra of pure CTAB and the products fabricated under different ingredient formulations.

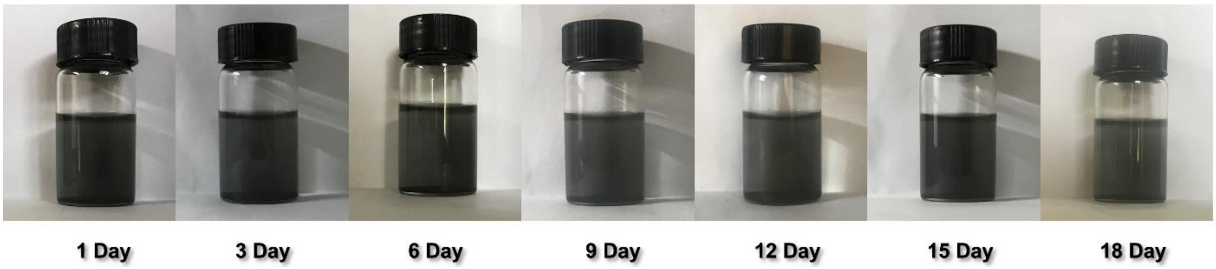

Figure S12. Optical photographs of aqueous solutions of HEA NPs at room temperature with different storage times.

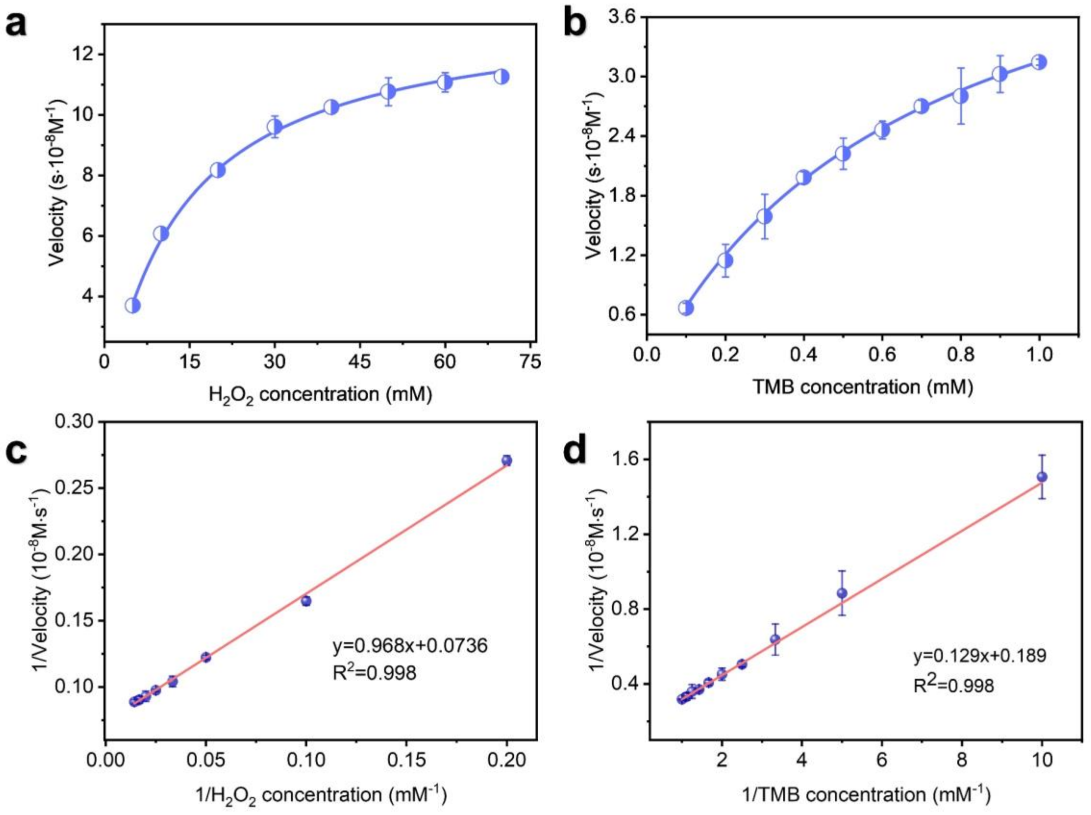

Figure S13. Enzymatic kinetics of aggregated HEA. (a, b) Michaelis-Menten curve of aggregated HEA (with only metal precursors) toward different concentrations of H_2O_2 and TMB, respectively. (c, d) Lineweaver-Burk double reciprocal curve plots for aggregated HEA with H_2O_2 and TMB. All data are presented as mean ± SD (n = 3 independent samples).

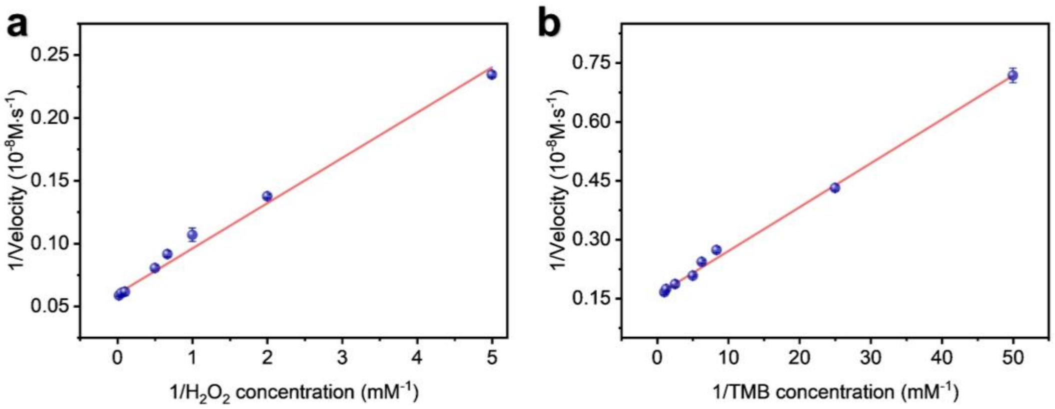

Figure S14. Enzymatic kinetics of HEA NPs. (a, b) Lineweaver-Burk double reciprocal curve plots for HEA NPs with H_2O_2 and TMB. All data are presented as mean ± SD (n = 3 independent samples).

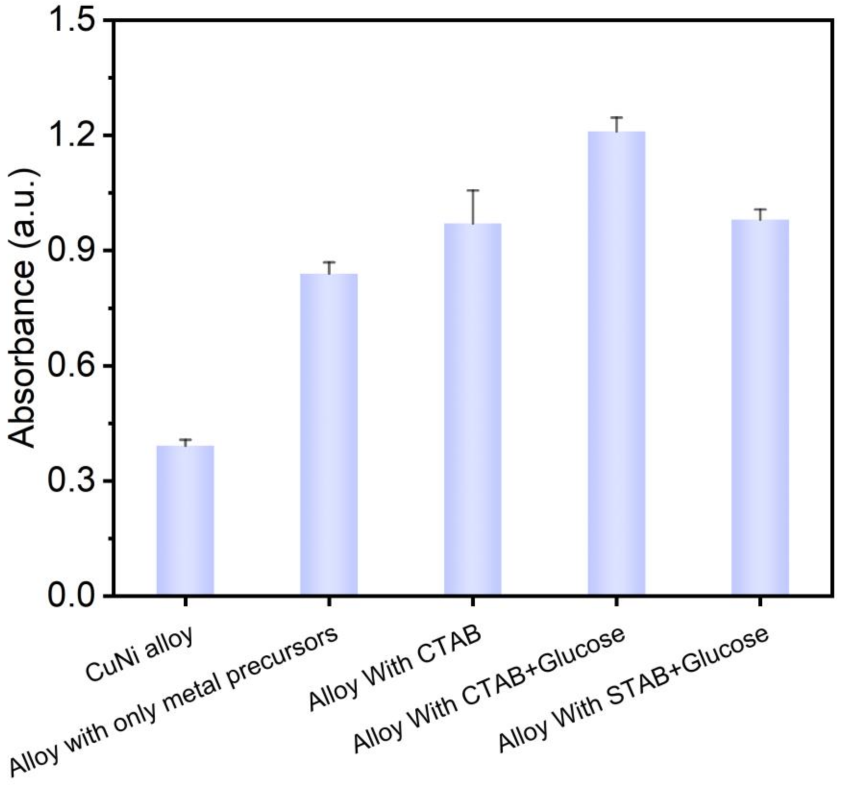

Figure S15. POD-like activity of the products synthesized under different ingredients formulation. CuNi alloys were obtained by mixing Cu and Ni metal precursors with CTAB and glucose, and heating in an oil bath at 220 °C. Other alloys were synthesized by using Mn, Fe, Co, Ni, and Cu metal precursors in various ingredient formulations under identical conditions. All data are presented as mean ± SD. All data are presented as mean ± SD (n = 3 independent samples).

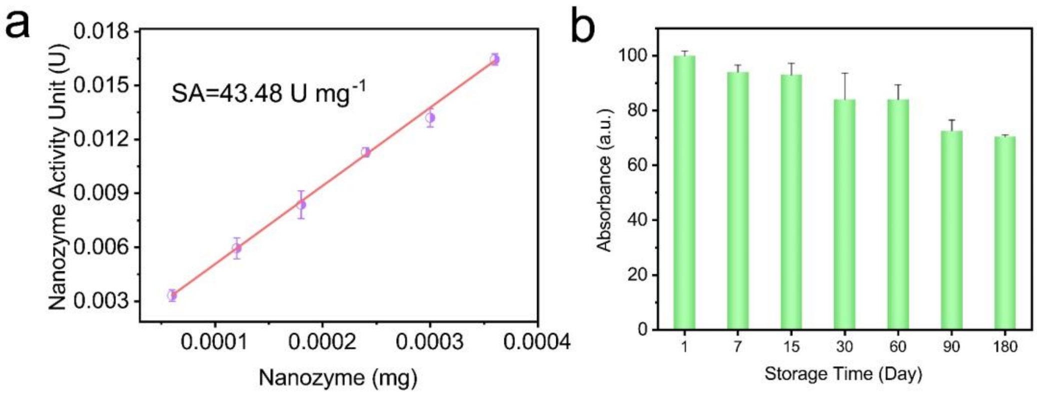

Figure S16. (a) The specific activity of the HEA bulk. (b) Variation of the POD-like activity of HEA NPs at room temperature with different storage times. The data are presented as the mean ± SD (n = 3 independent samples).

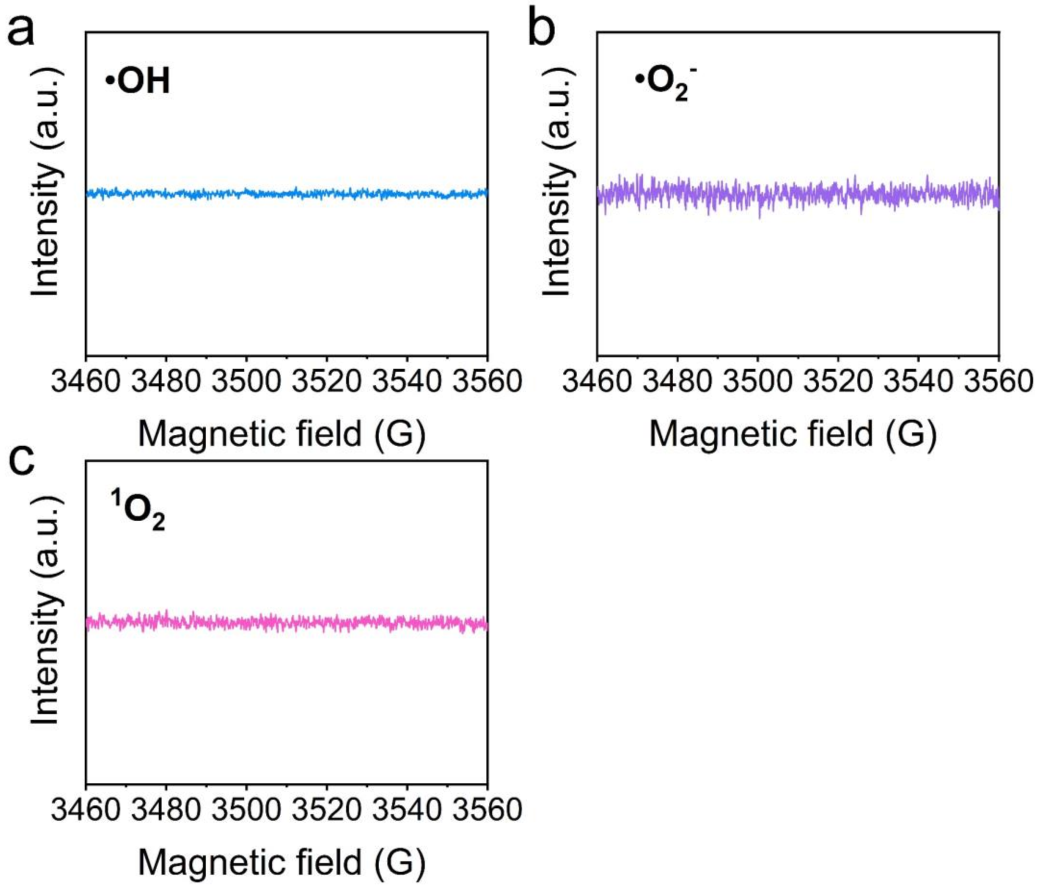

Figure S17. ESR spectra for the detection of (a) •OH, (b) $•O_2^-$, and (c) $^1O_2$ in the presence of HEA only.

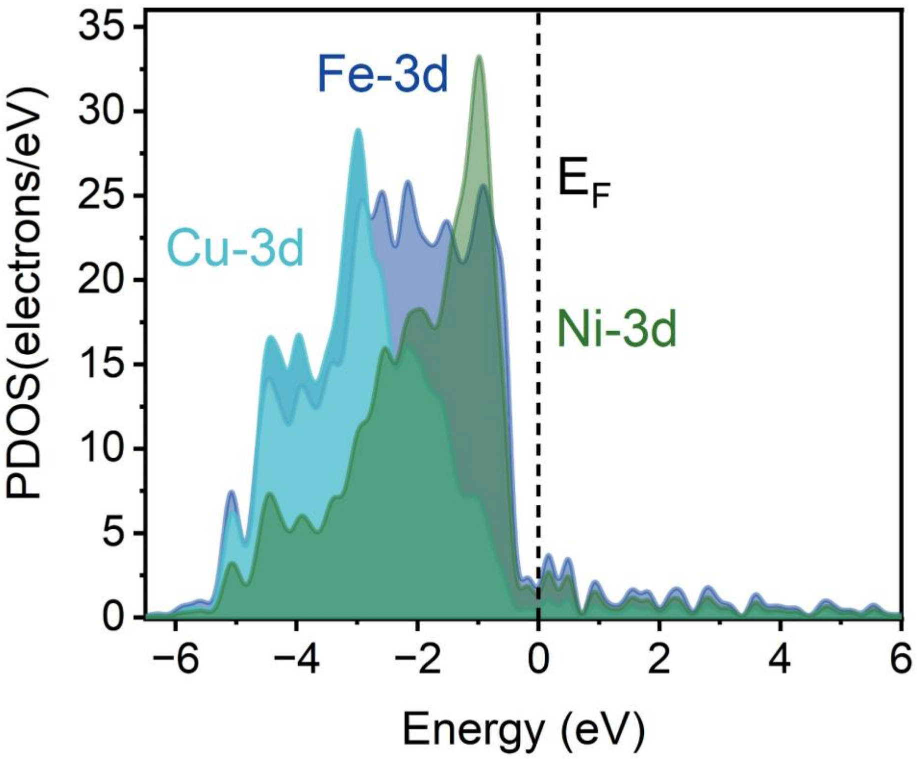

Figure S18. PDOS of the FeCuNi.

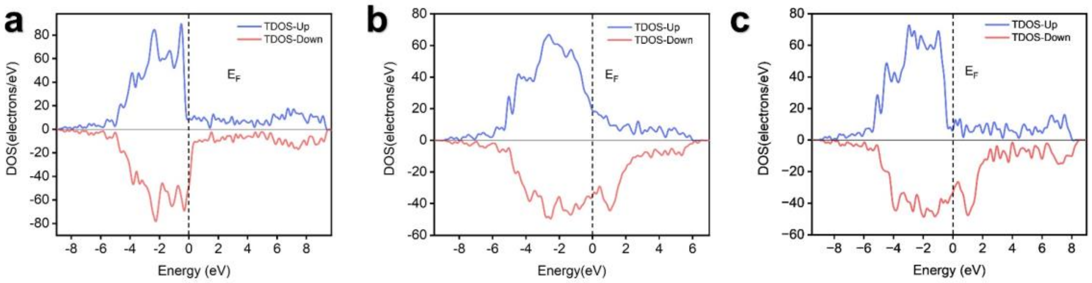

Figure S19. TDOS of (a) the CuNi, (b) HEA, and (c) FeCuNi.

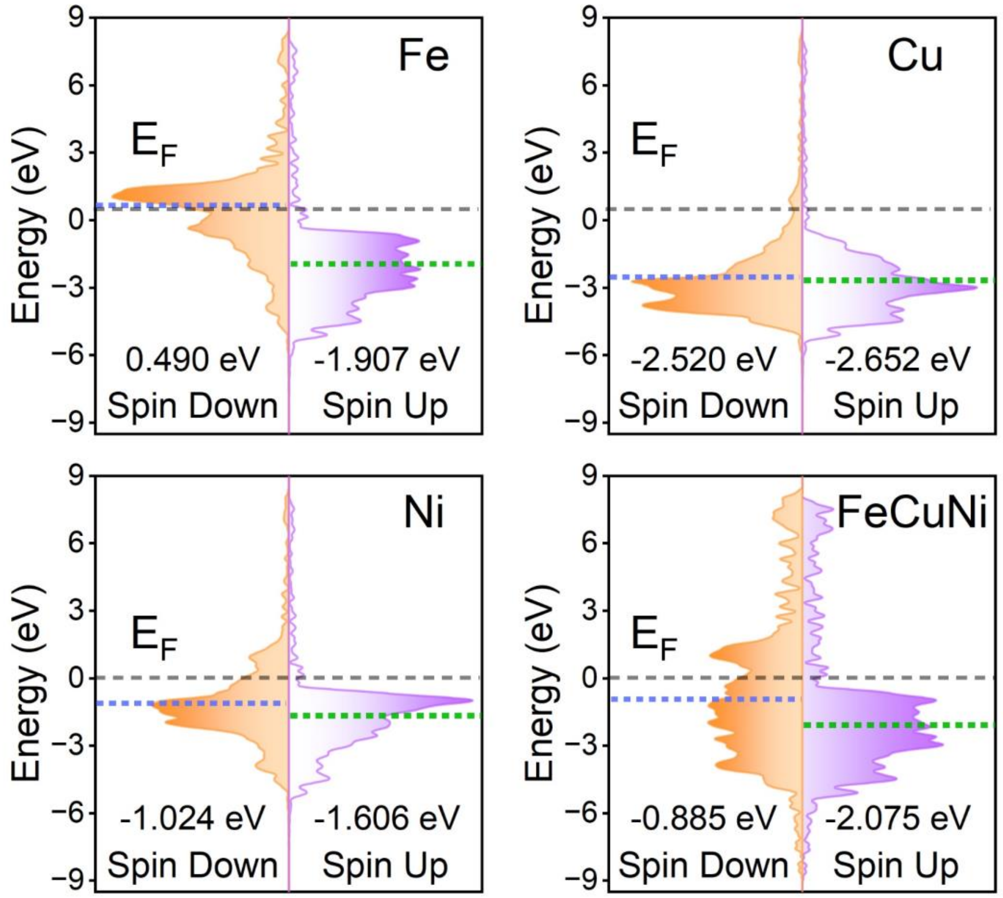

Figure S20. Calculated PDOSs and *d*-band centers (including spin-up and spin-down) for each element and the FeCuNi.

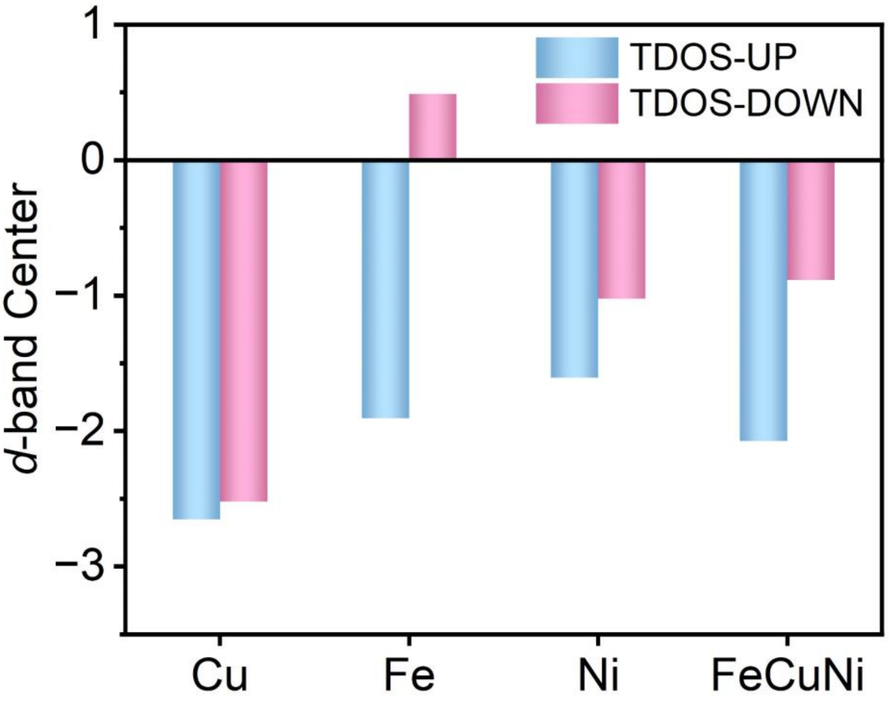

Figure S21. *d*-band center comparisons for the individual elements and the FeCuNi.

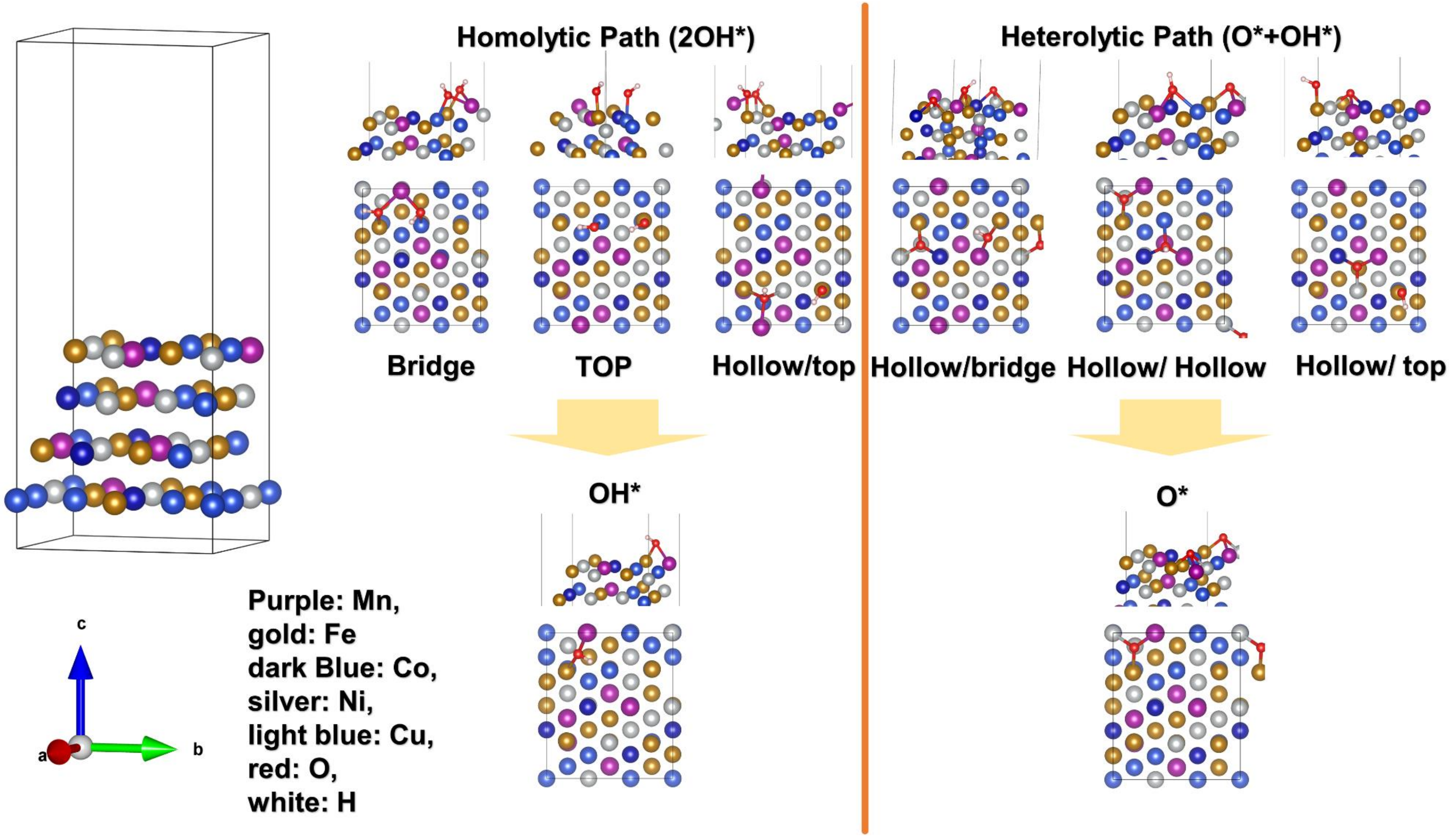

Figure S22. HEA model and the optimized oxygen-containing intermediate adsorption configurations on the surface of HEA.

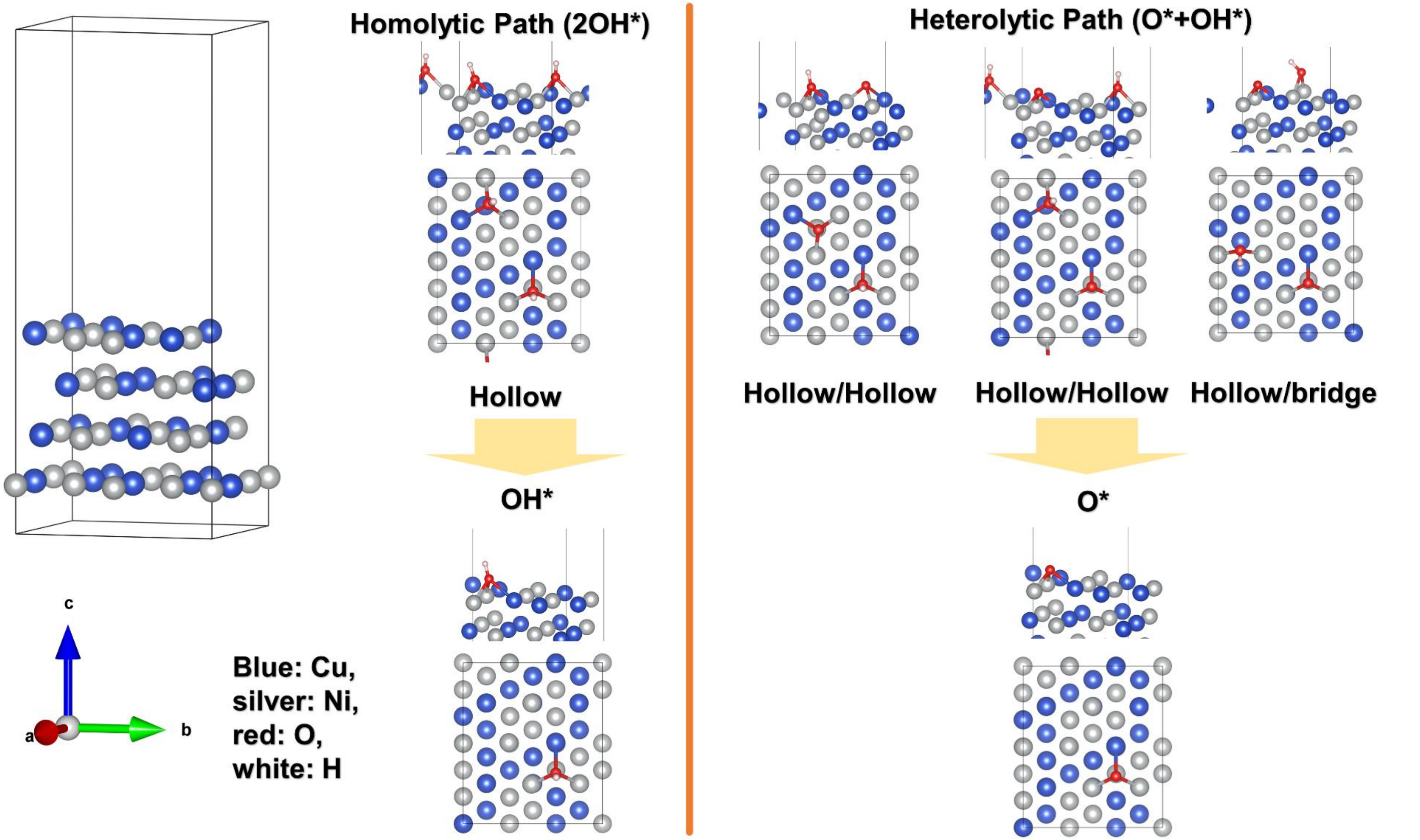

Figure S23. CuNi model and the optimized oxygen-containing intermediate adsorption configurations on the surface of CuNi.

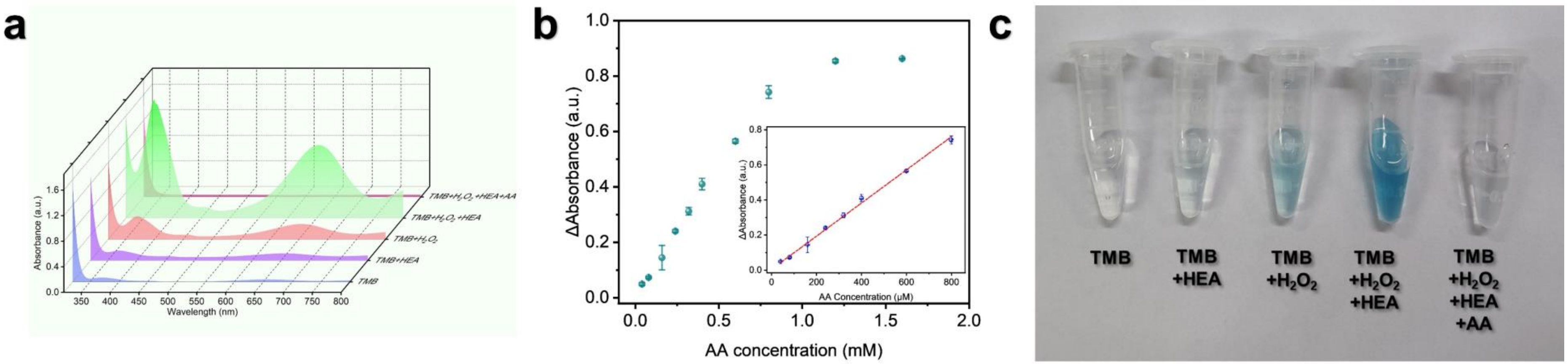

Figure S24. TAC assay based on HEA NPs. (a) UV-vis absorption spectra comparison for different reaction systems in TAC assay. (b) The AA-concentration dependence of HEA/H_2O_2/TMB system and linear calibration plot (inset image) of AA in the range 40-800 μM. Data are presented as mean ± SD (n = 3 independent samples). (c) Optical photograph comparison for different reaction systems in H_2O_2 and TAC biosensing.

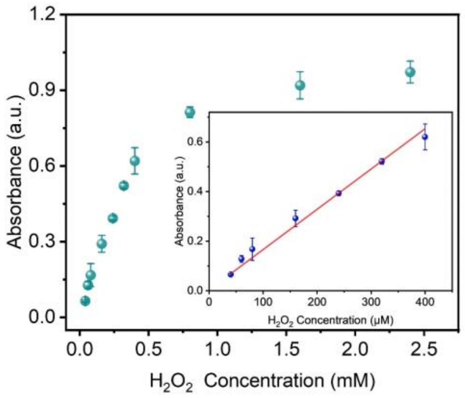

Figure S25. Colorimetric detection of H_2O_2 based on HEA NPs. The H_2O_2-concentration dependence of HEA NPs/TMB system and linear calibration plot (inset image) of H_2O_2 in the range 40 – 400 μM. Data are presented as mean ± SD (n = 3 independent samples).

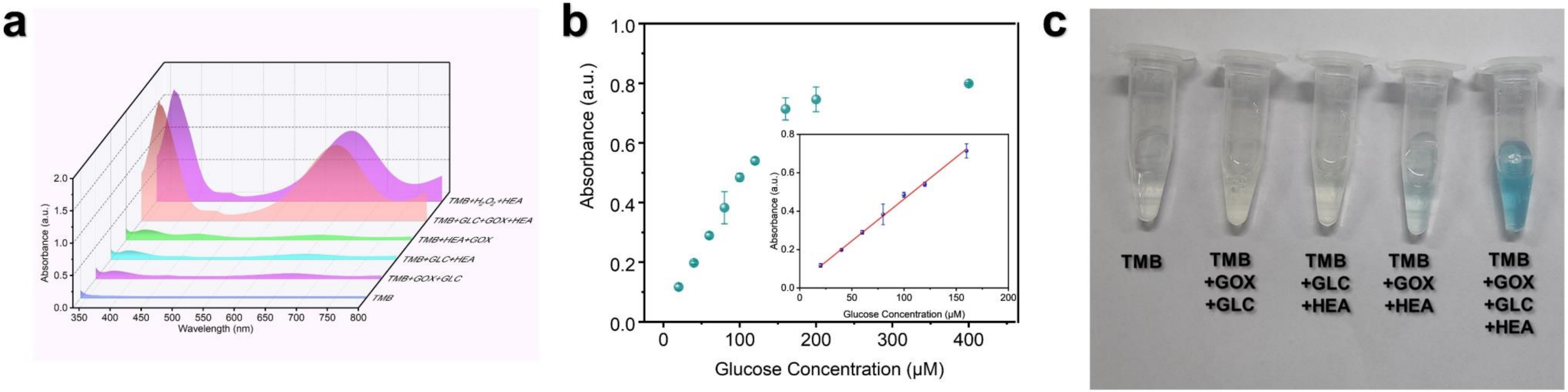

Figure S26. Colorimetric detection of glucose based on HEA NPs. (a) UV-vis absorption spectra comparison for different systems in glucose biosensing. (b) The glucose-concentration dependence of GOX/HEA/TMB system and linear calibration plot (inset image) of glucose in the range 20–160 μM. Data are presented as mean ± SD (n = 3 independent samples). (c) Optical photograph comparison for different reaction systems in glucose biosensing.

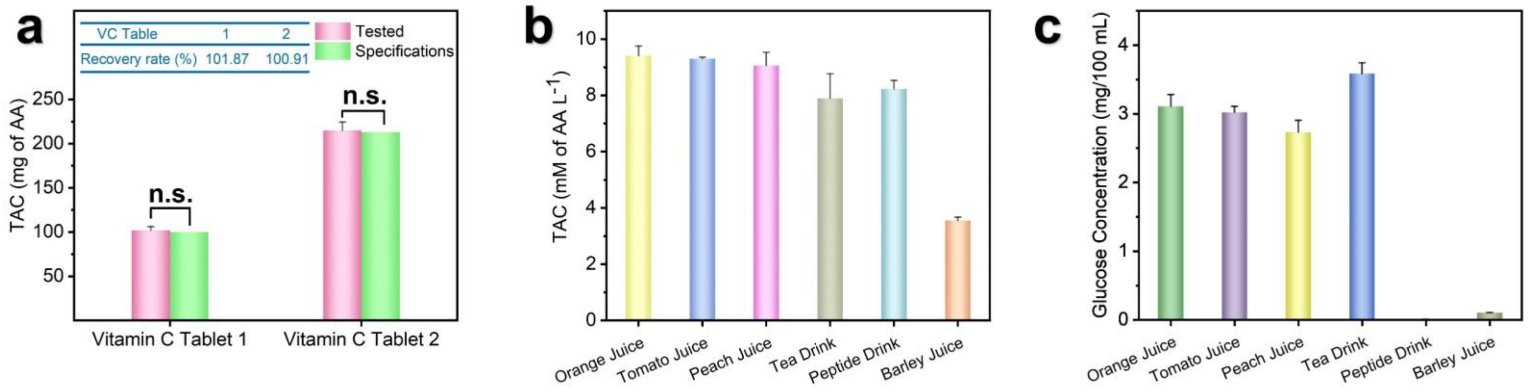

Figure S27. Sample testing of HEA NPs-based assays. (a) Verification test for the accuracy of TAC assay using vitamin C tablets. (b, c) Tested TAC values and glucose contents for commercial beverages. All data are presented as mean ± SD (n = 3 independent samples).

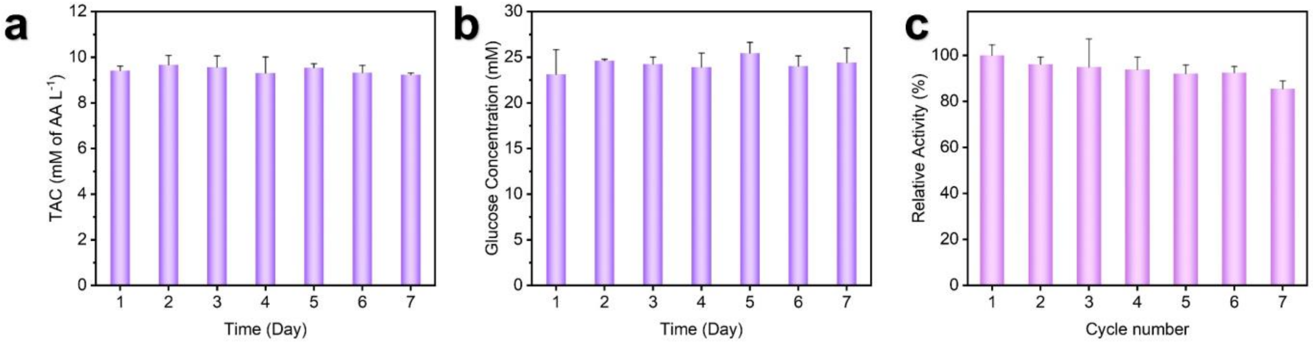

Figure S28. Stability testing of detection methods based on HEA NPs. (a, b) Stability of the established biosensors for TAC assay and glucose detection. (c) Reusability of the POD-like activity of HEA NPs. All data are presented as mean ± SD (n = 3 independent samples).

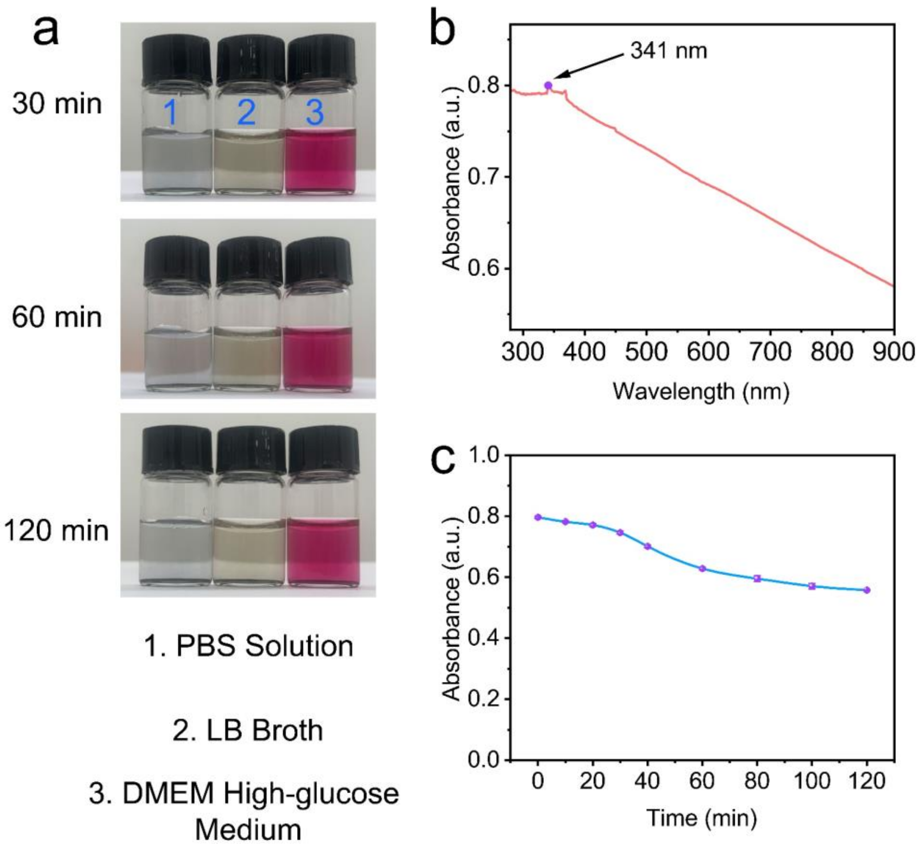

Figure S29. Dispersion test of HEA NPs. (a) Photographs of the dispersion of HEA NPs in different aqueous systems. (b) UV-vis absorption spectra of HEA NPs. c Curve of absorbance of HEA NPs PBS solution at 341 nm for different storage times. The data are presented as the mean ± SD (n = 3 independent samples).

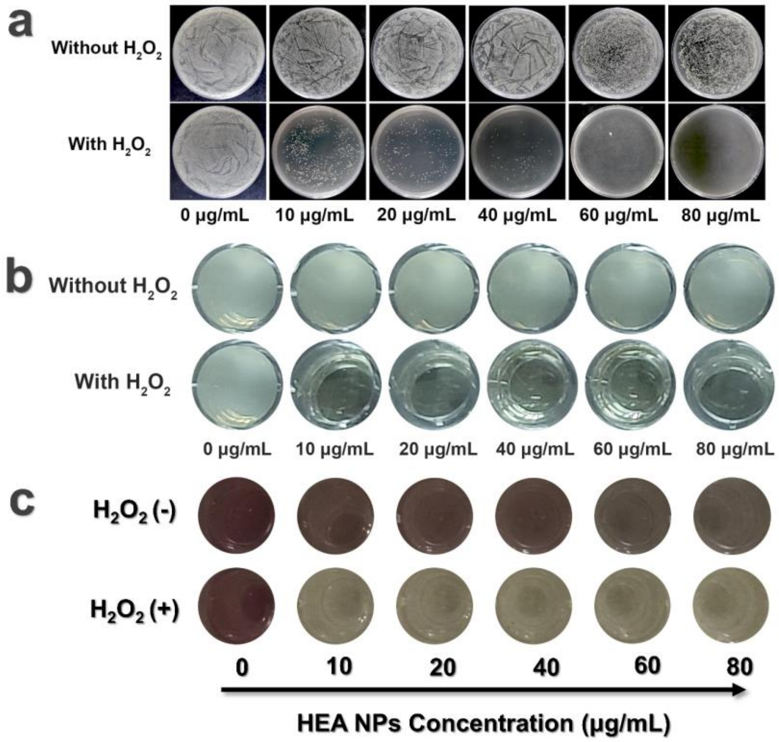

Figure S30. The antibacterial activity of HEA NPs/H_2O_2 system against MRSA. Photographs of (a) the bacterial colonies and (b) the turbidity of bacterial suspensions formed by MRSA after different treatments. (c) Photograph of the determination of the bactericidal capacity of the HEA/H_2O_2 system against MRSA by MTT assay.

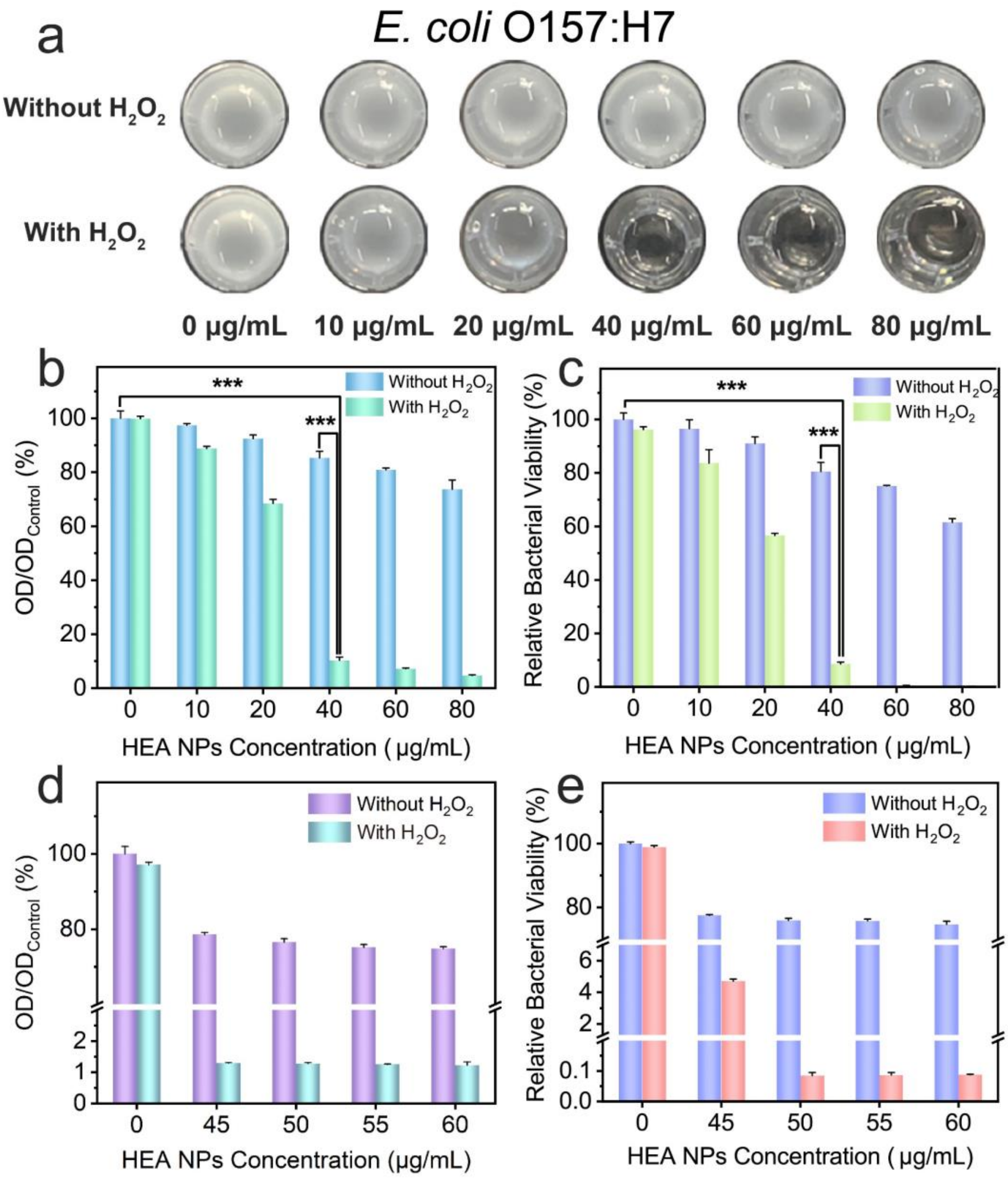

Figure S31. Determination of MIC and MBC of HEA NPs/H_2O_2 antibacterial system against *E. coli* O157:H7. (a) Turbidity of bacterial suspensions formed by *E. coli* O157:H7 after different treatments. (b) Evaluation of the inhibitory effect of different treatments on the growth of *E. coli* O157:H7 after incubation. The data are presented as the mean ± SD (n = 3 independent samples). (c) Determination of the inhibitory effect of HEA/H_2O_2 system on *E. coli* O157:H7 by MTT assay. The data are presented as the mean ± SD (n = 3 independent samples). (d) Evaluation of the inhibitory effect of the HEA NPs/H_2O_2 system on the growth of *E. coli* O157:H7 at doses near the MIC. The data are presented as the mean ± SD (n = 3 independent samples). (e) Determination

of the bactericidal efficiency of the HEA/H_2O_2 system against *E. coli* O157:H7 by MTT assay. The data are presented as the mean ± SD (n = 3 independent samples). One-way ANOVA and one-sided Tukey's multiple comparison test were performed to evaluate the differences in the means of the groups (significance level: ***P<0.001).

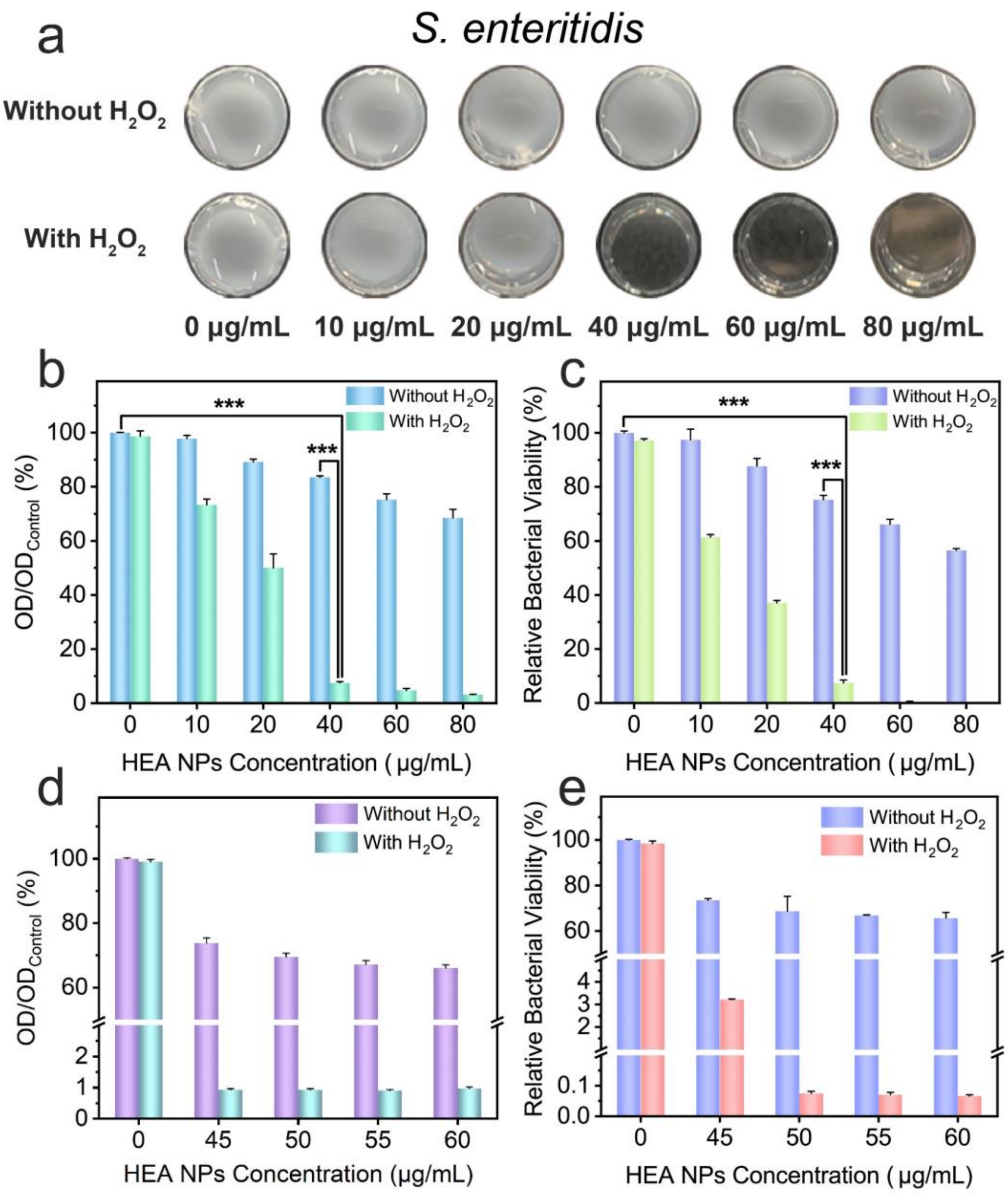

Figure S32. Determination of MIC and MBC of HEA NPs/H_2O_2 antibacterial system against *S. enteritidis*. (a) Turbidity of bacterial suspensions formed by *S. enteritidis* after different treatments. (b) Evaluation of the inhibitory effect of different treatments on the growth of *S. enteritidis* after incubation. The data are presented as the mean ± SD (n = 3 independent samples). (c) Determination of the inhibitory effect of HEA/H_2O_2 system on *S. enteritidis* by MTT assay. The data are presented as the mean ± SD (n = 3 independent samples). (d) Evaluation of the inhibitory effect of the HEA NPs/H_2O_2 system on the growth of *S. enteritidis* at doses near the MIC. The data are presented as the mean ± SD (n = 3 independent samples). (e) Determination of the bactericidal

efficiency of the HEA/H_2O_2 system against *S. enteritidis* by MTT assay. The data are presented as the mean ± SD (n = 3 independent samples). One-way ANOVA and one-sided Tukey's multiple comparison test were performed to evaluate the differences in the means of the groups (significance level: ***P<0.001).

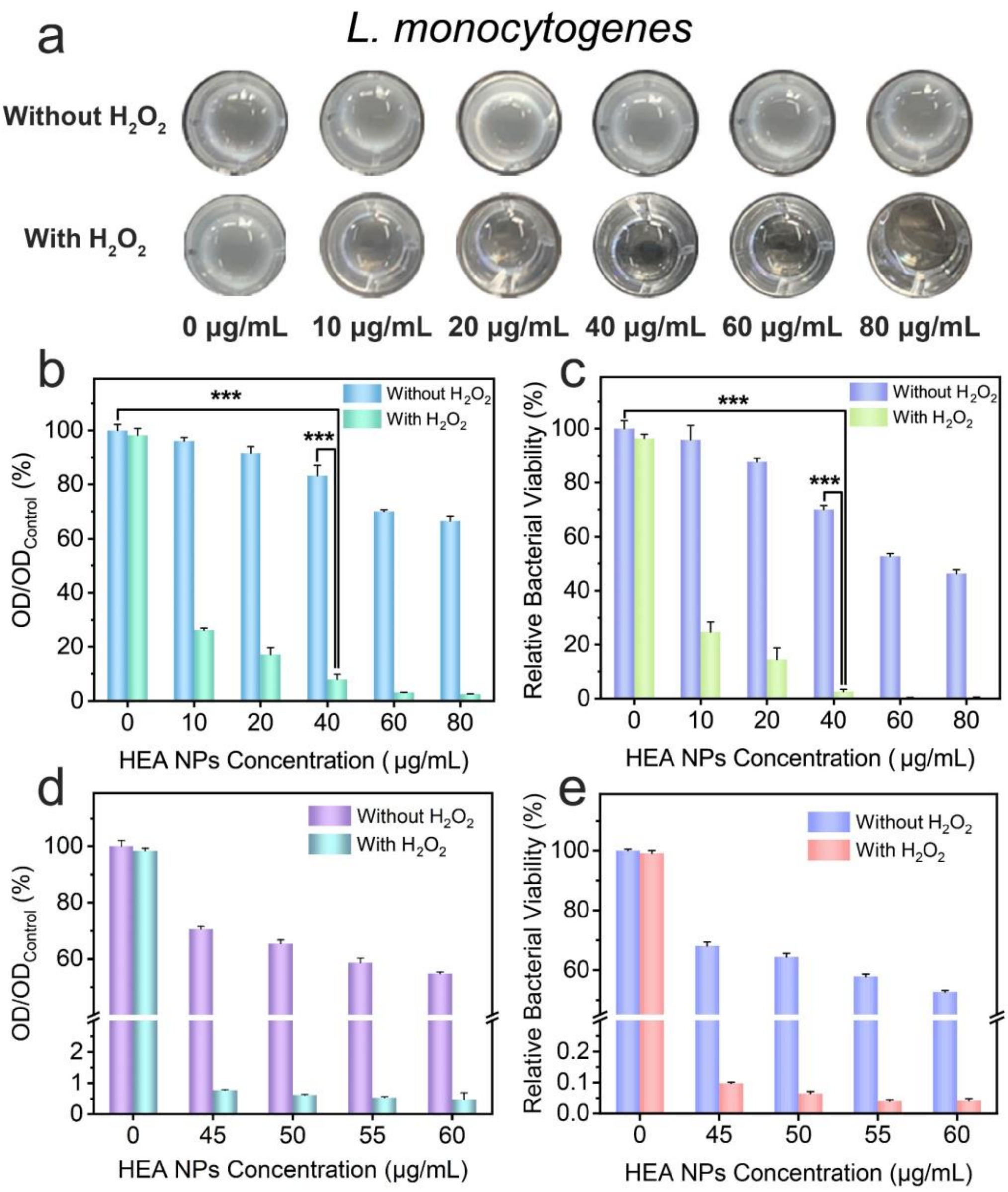

Figure S33. Determination of MIC and MBC of HEA NPs/H_2O_2 antibacterial system against *L. monocytogenes*. (a) Turbidity of bacterial suspensions formed by *L. monocytogenes* after different treatments. (b) Evaluation of the inhibitory effect of different treatments on the growth of *L. monocytogenes* after incubation. The data are presented as the mean ± SD (n = 3 independent samples). (c) Determination of the inhibitory effect of HEA/H_2O_2 system on *S. enteritidis* by MTT assay. The data are presented as the mean ± SD (n = 3 independent samples). (d) Evaluation of the inhibitory effect of the HEA NPs/H_2O_2 system on the growth of *L. monocytogenes* at doses near the MIC. The data are presented as the mean ± SD (n = 3 independent

samples). (e) Determination of the bactericidal efficiency of the HEA/H_2O_2 system against *L. monocytogenes* by MTT assay. The data are presented as the mean ± SD (n = 3 independent samples). One-way ANOVA and one-sided Tukey's multiple comparison test were performed to evaluate the differences in the means of the groups (significance level: ****P*<0.001).

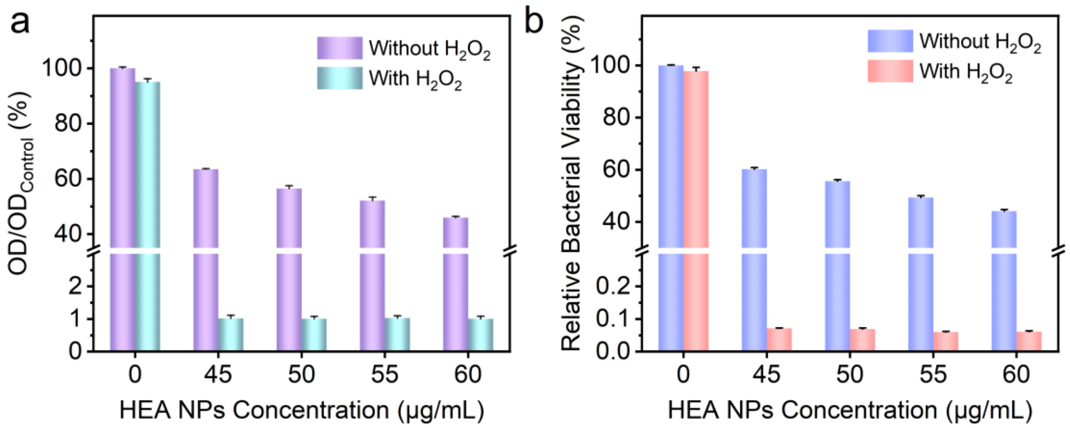

Figure S34. Determination of MBC of HEA NPs/H_2O_2 antibacterial system against MRSA. (a) Evaluation of the inhibitory effect of the HEA NPs/H_2O_2 system on the growth of MRSA at doses near the MIC. The data are presented as the mean ± SD (n = 3 independent samples). (b) Determination of the bactericidal efficiency of the HEA/H_2O_2 system against MRSA by MTT assay. The data are presented as the mean ± SD (n = 3 independent samples).

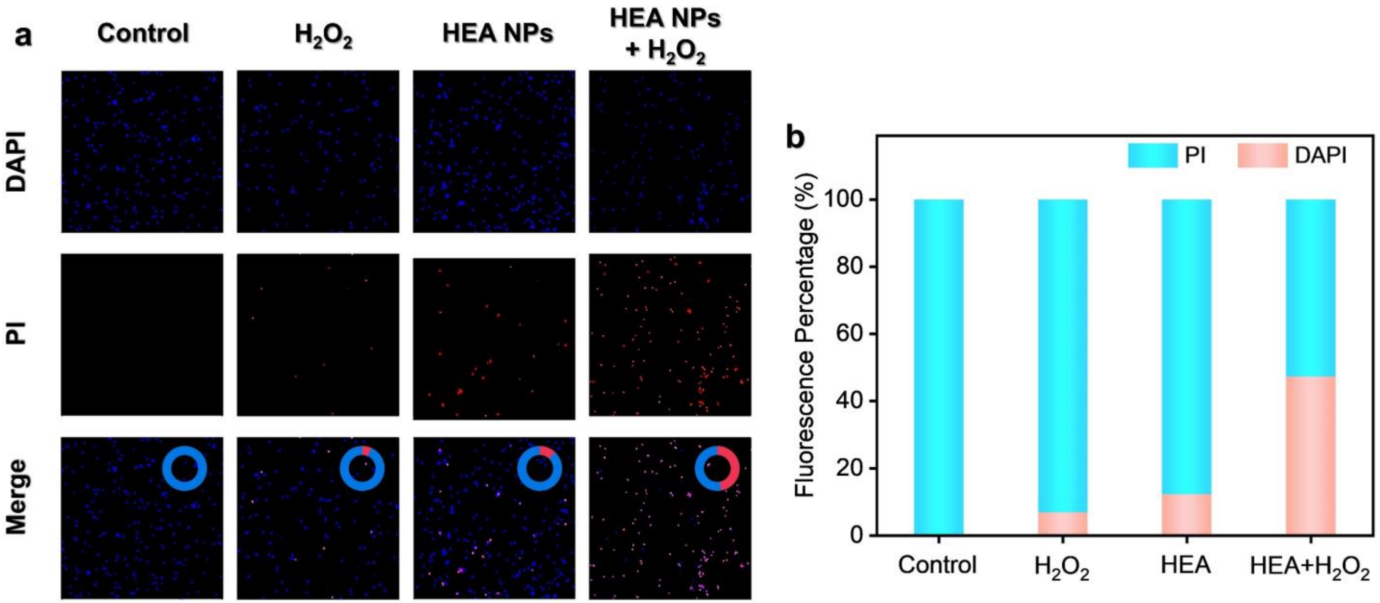

Figure S35. Live/dead bacterial staining. (a) CLSM images of MRSA stained with DAPI/PI after different processing. (b) Quantitative analysis results of fluorescence intensity from live/dead bacteria staining (DAPI/PI) fluorescent probe.

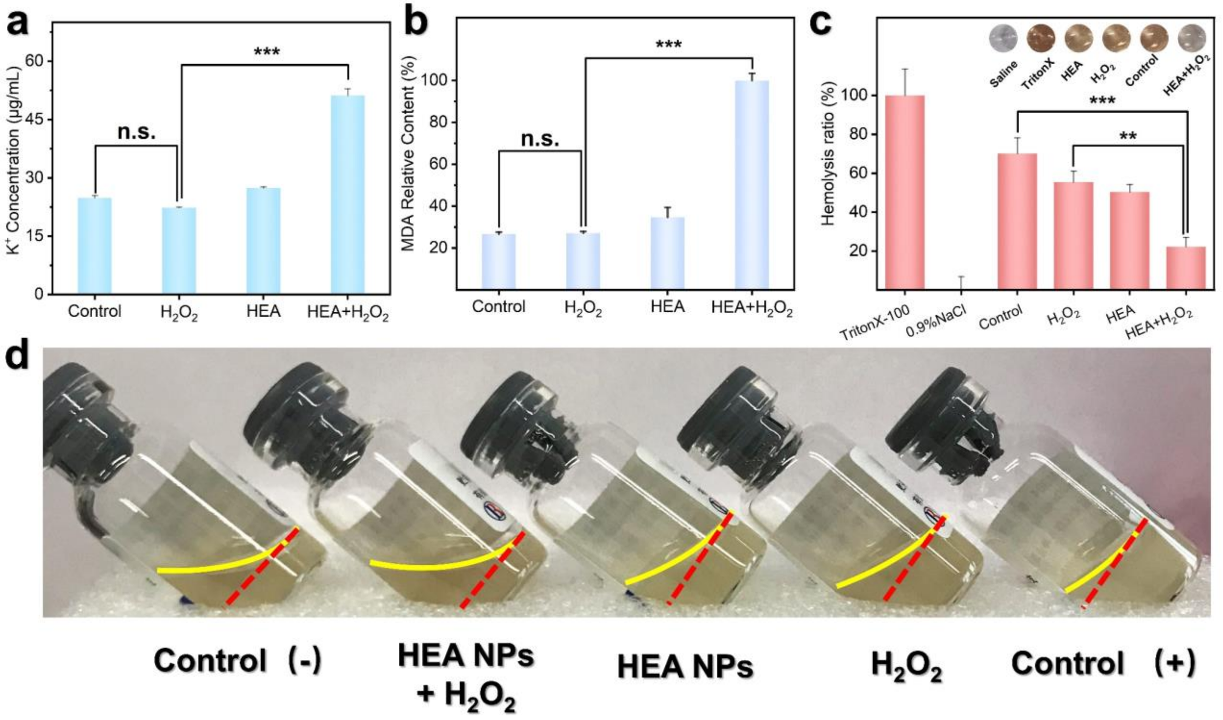

Figure S36. Study on antibacterial mechanism of HEA NPs/H_2O_2 system. (a) Leakage of bacterial intracellular K^+. (b) Levels of lipid peroxidation in bacteria after different treatments. (c) Effect of different treatments on the hemolytic capacity of MRSA. All data are presented as mean ± SD (n = 3 independent samples, significance level: $^{**}P<0.01$, $^{***}P<0.001$). (d) Optical photograph of the plasma coagulase test for bacterial suspensions with different treatments.

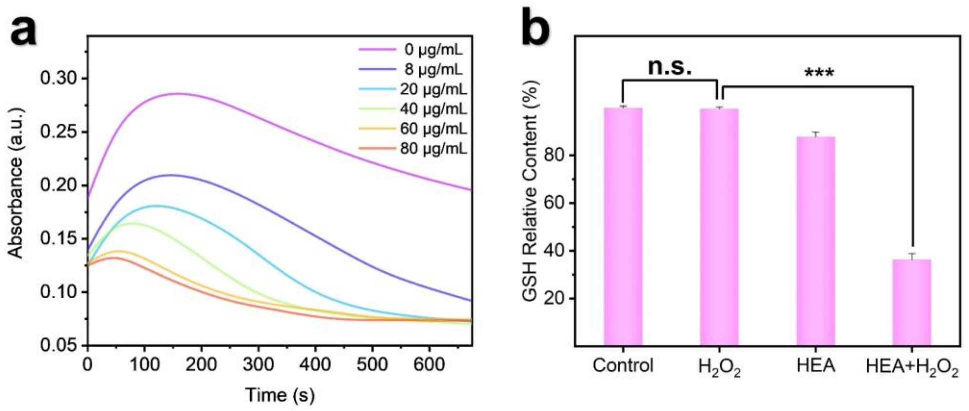

Figure S37. GSH depletion capacity of HEA NPs. (a) Catalyst concentration-dependent GSH consumption curve. (b) Depletion of GSH from MRSA under different treatments. Data are presented as mean ± SD (n = 3 independent samples, significance level: ****P*<0.001).

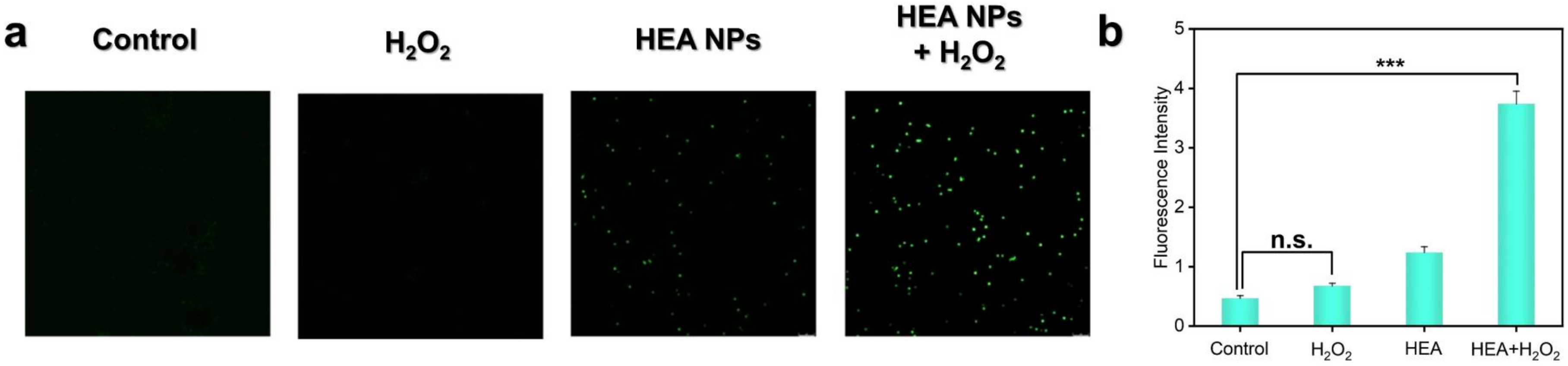

Figure S38. Detection of endogenous ROS in bacteria. (a) CLSM images and (b) the corresponding quantitative analysis results using DCFH-DA fluorescent probe. Data are presented as mean ± SD (n = 3 independent samples, significance level: ***P<0.001).

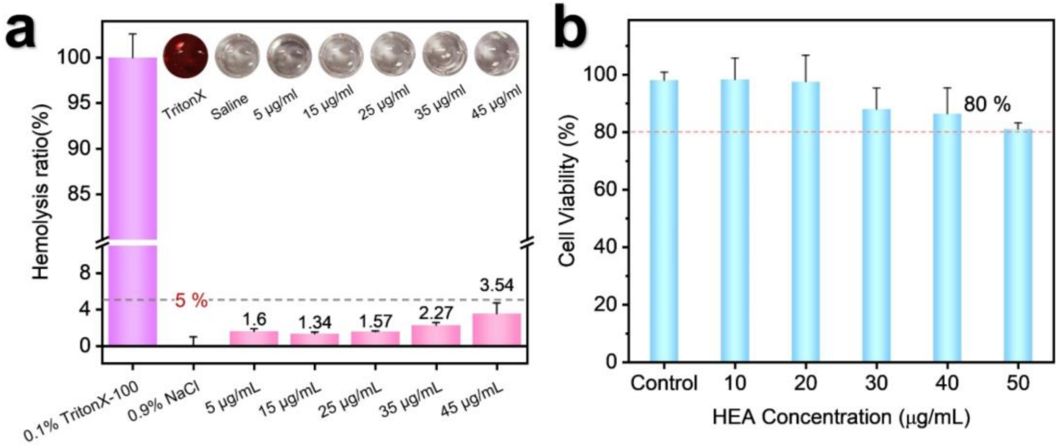

Figure S39. Biocompatibility of HEA nanozymes. (a) Hemolysis assay of HEA NPs at various concentrations. The inset image illustrates the color of the supernatant after the test. (b) Cell viability of 3T3 cells after incubation with HEA NPs. All data are presented as mean ± SD (n = 3 independent samples).

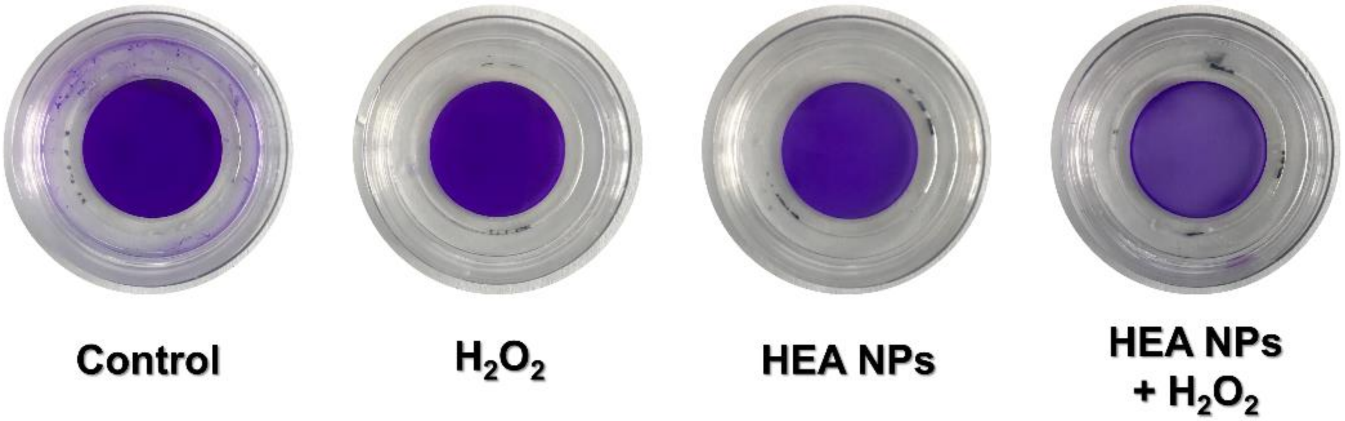

Figure S40. Optical photographs of crystal violet staining results of MRSA biofilms after different treatments (dissolved with 33% acetic acid aqueous solution).

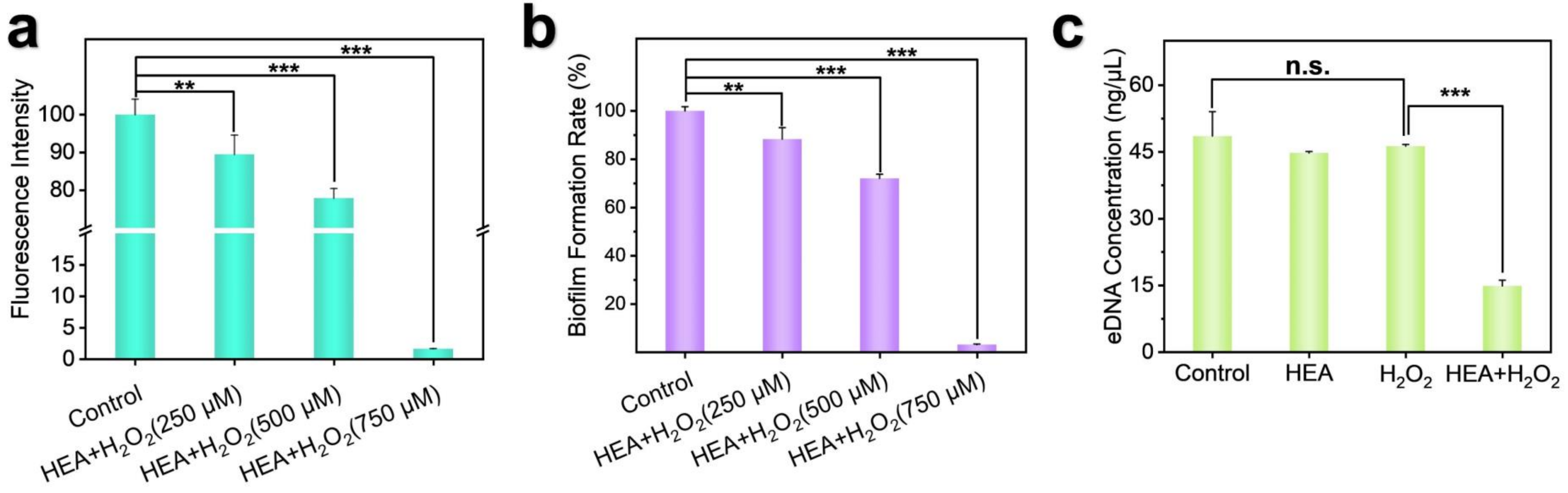

Figure S41. Evaluation of the anti-biofilm activity of HEA NPs/H_2O_2 system. (a) The fluorescence intensities for MRSA biofilm formation in 3D CLSM images. (b) Crystal violet staining results of MRSA biofilms. The data are presented as the mean ± SD (n = 3 independent samples). (c) Comparison of the extracellular DNA levels in biofilms after different treatments. The data are presented as the mean ± SD (n = 3 independent samples). The data are presented as the mean ± SD (n = 3 independent samples). One-way ANOVA and one-sided Tukey's multiple comparison test were performed to evaluate the differences in the means of the groups (significance level: ***P*<0.01, ****P*<0.001).

Table S1. Enzyme kinetic parameters of HEA NPs as POD mimics.

Nannozyme	Substrate	K_m (mM)	V_{max} (10^{-8} M s^{-1})	Refs.
HEA NPs	H_2O_2	0.60	16.62	This work
	TMB	0.07	6.26	
Aggregated HEA (with only metal precursors)	H_2O_2	13.18	13.59	This work
	TMB	0.68	5.28	
HRP	H_2O_2	3.70	8.71	[1]
	TMB	0.43	10.00	

Table S2. Comparison of the kinetic parameters of various POD mimics.

POD mimics	[E]/M	Substrate	K_m(mM)	V_{max} (10^{-8} M s^{-1})	K_{cat} (min^{-1})	K_{cat}/K_m (M^{-1} min^{-1})	Refs.
HEA NPs	3.09×10^{-14}	H_2O_2	0.60	16.62	3.23×10^{8}	5.38×10^{11}	This work
		TMB	0.07	6.26	1.22×10^{8}	1.74×10^{12}	
Fe_3O_4 MNPs	11.4×10^{-13}	H_2O_2	154	9.78	5.15×10^{6}	5.57×10^{5}	[1]
		TMB	0.098	3.44	1.81×10^{6}	1.85×10^{10}	
ISPtNP	8.12×10^{-11}	H_2O_2	769	126	9.3×10^{5}	1.21×10^{6}	[2]
		TMB	0.120	185	1.36×10^{6}	1.14×10^{10}	
PMCS	2.15×10^{-14}	H_2O_2	40.16	12.15	3.39×10^{8}	8.46×10^{9}	[3]
		TMB	0.224	10.66	2.98×10^{8}	1.33×10^{12}	
$ZnFe_2O_4$ MNPs	3.05×10^{-18}	H_2O_2	1.66	7.74	1.52×10^{12}	9.18×10^{11}	[4]
		TMB	0.85	13.31	2.62×10^{12}	3.08×10^{12}	
PBNPs	1.96×10^{-12}	H_2O_2	14.7	11.5	3.52×10^{6}	2.40×10^{8}	[5]
	1.86×10^{-12}	TMB	0.337	21.6	6.96×10^{6}	2.07×10^{10}	
HRP	2.5×10^{-11}	H_2O_2	3.70	8.71	2.09×10^{5}	5.64×10^{7}	[1]
		TMB	0.434	10	2.40×10^{5}	5.53×10^{8}	

Table S3. Glucose detection results in different beverage samples.

Sample	Orange juice	Peach juice	Tomato juice	Tea juice	Peptide drink	Barley juice
Recovery rate (%)	98.68	97.52	95.88	105.61	95.68	102.16

Table S4. Comparison of various nanozymes serving as TAC biosensors.

Nanozyme	Linear range (AA equivalent)	Limit of detection (AA equivalent)	Refs.
HEA NPs	40-800 μM	28.59 μM	This work
CNT/FeNC	0.1-10 μM	0.03 μM	[6]
Fe–N–C SANs	0.5-33 μM	0.5 μM	[7]
SNC nanozymes	0.1-5 mM	80 μM	[8]
CP_{600-6}	8-80 μM	8.0 μM	[9]
Dex-FeMnzyme	1-30 μM	1.17 μM	[10]
Pt/CeO_2 nanocomposites	0.5-30 μM	80 nM	[11]

Table S5. Comparison of various nanozymes serving as glucose biosensors.

Nanozyme	Linear range	Limit of detection	Refs.
HEA NPs	20-160 μM	4.00 μM	This work
CNT/FeNC	0.1-10 mM	20 μM	[6]
GOx@ZIF-8(NiPd)	0.01-0.3 mM	9.2 μM	[12]
CP_{600-6}	10-100 μM	10 μM	[9]
B-GDY	50-400 μM	-	[13]
AuNPs-O-g-C_3N_4- ABEI	0.5-100 μM	0.1 μM	[14]
GOx@Co-m-ceria	0.1-1.5 mM	7 μM	[15]

Table S6. MIC and MBC of HEA NPs/H_2O_2 antibacterial system against different bacterial strains.

Bacterial strain	Gram bacteria classification	Minimum inhibitory concentration (MIC, μg/mL)	Minimum bactericidal concentration (MBC, μg/mL)
MRSA	Gram-positive	40	45
L. monocytogenes	Gram-positive	40	45
E. coli O157:H7	Gram-negative	40	50
S. enteritidis	Gram-negative	40	50

Table S7. Comparison of various nanozymes for antibacterial or antibiofilm applications.

Nanozyme	Types of enzyme mimics	Domain of application	Mechanism of action	Effective dose of nanozyme	Species of test bacteria	Refs.
HEA NPs	POD	Antibacterial	ROS-mediated	40 µg/mL<br>H_2O_2: 200 µM	MRSA	This work
N-CNTs@Co	OXD	Antibacterial	ROS-mediated	60 µg/mL	*S. aureus*	[16]
Apt-PtNZs	POD	Antibacterial	ROS-mediated and GOX-assist	30 µg/mL<br>H_2O_2: 1 mM	*S. aureus*	[17]
PMCS	POD	Antibacterial	ROS-mediated	100 µg/mL<br>H_2O_2: 100 µM	*P. aeruginosa*	[3]
CuS@GDY	POD	Antibacterial	ROS-mediated with photothermal synergy	100 µg/mL<br>H_2O_2: 100 µM	*S. aureus*	[18]
Ag/BMO NPs	POD	Antibacterial	ROS-mediated with photodynamic-assistance	200 µg/mL<br>H_2O_2: 3 mM	*MRSA*	[19]
FeN_5 SA/CNF	OXD	Antibacterial	ROS-mediated	100 µg/mL	*S. aureus*	[20]

Nanozyme	Types of enzyme mimics	Domain of application	Mechanism of action	Effective dose of nanozyme	Species of test bacteria	Refs.
Pt_{TS}-SAzyme	POD	Antibacterial	ROS-mediated	250 μg/mL<br>H_2O_2: 1 mM	*E. coli*	[21]
PtCo@G@CPB	OXD	Antibacterial	ROS-mediated	70 μg/mL	*H. pylori*	[22]
CS@Fe/CDs	POD	Antibiofilm	ROS-mediated	50 μg/mL<br>H_2O_2: 500 μM	*P. aeruginosa*	[23]
Ce-FMA-FA-20-RT	Hydrolases	Antibiofilm	Hydrolysis	50 μg/mL	*E. coli*	[24]
[Fe(TPP)]Cl	Polyzymes	Antibiofilm	Assistance for targeted release of antibiotics	200 nM	*E. coli*	[25]

References

[1] L. Gao, J. Zhuang, L. Nie, J. Zhang, Y. Zhang, N. Gu, T. Wang, J. Feng, D. Yang, S. Perrett, X. Yan, *Nat. Nanotechnol.* **2007**, *2* (9), 577.

[2] Z. Gao, M. Xu, L. Hou, G. Chen, D. Tang, *Anal. Chim. Acta* **2013**, *776*, 79.

[3] B. Xu, H. Wang, W. Wang, L. Gao, S. Li, X. Pan, H. Wang, H. Yang, X. Meng, Q. Wu, L. Zheng, S. Chen, X. Shi, K. Fan, X. Yan, H. Liu, *Angew. Chem. Int. Ed. Engl.* **2019**, *58* (15), 4911.

[4] L. Su, J. Feng, X. Zhou, C. Ren, H. Li, X. Chen, *Anal. Chem.* **2012**, *84* (13), 5753.

[5] W. Zhang, S. Hu, J. J. Yin, W. He, W. Lu, M. Ma, N. Gu, Y. Zhang, *J. Am. Chem. Soc.* **2016**, *138* (18), 5860.

[6] N. Cheng, J. C. Li, D. Liu, Y. Lin, D. Du, *Small* **2019**, *15* (48), e1901485.

[7] L. Shen, M. A. Khan, X. Wu, J. Cai, T. Lu, T. Ning, Z. Liu, W. Lu, D. Ye, H. Zhao, J. Zhang, *Biosens. Bioelectron.* **2022**, *205*, 114097.

[8] Y. Chen, L. Jiao, H. Yan, W. Xu, Y. Wu, H. Wang, W. Gu, C. Zhu, *Anal. Chem.* **2020**, *92* (19), 13518.

[9] Z. Lou, S. Zhao, Q. Wang, H. Wei, *Anal. Chem.* **2019**, *91* (23), 15267.

[10] X. Han, L. Liu, H. Gong, L. Luo, Y. Han, J. Fan, C. Xu, T. Yue, J. Wang, W. Zhang, *Food Chem.* **2022**, *371*, 131115.

[11] X. Liu, X. Wang, C. Qi, Q. Han, W. Xiao, S. Cai, C. Wang, R. Yang, *Appl. Surf. Sci.* **2019**, *479*, 532.

[12] Q. Wang, X. Zhang, L. Huang, Z. Zhang, S. Dong, *Angew. Chem. Int. Ed. Engl.* **2017**, *56* (50), 16082.

[13] H. Qi, Y. Tong, M. Zhang, X. Wu, L. Yue, *Anal. Chem.* **2022**, *94* (49), 17272.

[14] Y. Gao, Y. Huang, J. Chen, Y. Liu, Y. Xu, X. Ning, *Anal. Chem.* **2021**, *93* (30), 10593.

[15] P. T. Nguyen, J. Lee, A. Cho, M. S. Kim, D. Choi, J. W. Han, M. I. Kim, J. Lee, *Adv. Funct. Mater.* **2022**, *32* (21).

[16] S. Y. He, J. Q. Huang, Q. Zhang, W. Zhao, Z. A. Xu, W. Zhang, *Adv. Funct. Mater.* **2021**, *31* (41).

[17] L. Chen, S. Xing, Y. Lei, Q. Chen, Z. Zou, K. Quan, Z. Qing, J. Liu, R. Yang, *Angew. Chem. Int. Ed. Engl.* **2021**, *60* (44), 23534.

[18] Q. Bai, M. Liang, W. Wu, C. Zhang, X. Li, M. Liu, D. Yang, W. W. Yu, Q. Hu, L. Wang, F. Du, N. Sui, Z. Zhu, *Adv. Funct. Mater.* **2022**, *32* (20).

[19] C. Cao, T. Zhang, N. Yang, X. Niu, Z. Zhou, J. Wang, D. Yang, P. Chen, L. Zhong, X. Dong, Y. Zhao, *Signal. Transduct. Target Ther.* **2022**, *7* (1), 86.

[20] L. Huang, J. Chen, L. Gan, J. Wang, S. Dong, *Sci. Adv.* **2019**, *5* (5), eaav5490.

[21] Y. Chen, P. Wang, H. Hao, J. Hong, H. Li, S. Ji, A. Li, R. Gao, J. Dong, X. Han, M. Liang, D. Wang, Y. Li, *J. Am. Chem. Soc.* **2021**, *143* (44), 18643.

[22] L. Zhang, L. Zhang, H. Deng, H. Li, W. Tang, L. Guan, Y. Qiu, M. J. Donovan, Z. Chen, W. Tan, *Nat. Commun.* **2021**, *12* (1), 2002.

[23] T. Pan, H. Chen, X. Gao, Z. Wu, Y. Ye, Y. Shen, *J. Hazard. Mater.* **2022**, *435*, 128996.

[24] S. Li, Z. Zhou, Z. Tie, B. Wang, M. Ye, L. Du, R. Cui, W. Liu, C. Wan, Q. Liu, S. Zhao, Q. Wang, Y. Zhang, S. Zhang, H. Zhang, Y. Du, H. Wei, *Nat. Commun.* **2022**, *13* (1), 827.

[25] R. Huang, C. H. Li, R. Cao-Milan, L. D. He, J. M. Makabenta, X. Zhang, E. Yu, V. M. Rotello, *J. Am. Chem. Soc.* **2020**, *142* (24), 10723.
